# The SLC15A4–TASL complex is essential for lupus development in mice

**DOI:** 10.1073/pnas.2618215123

**Published:** 2026-09-01

**Authors:** Ales Drobek, Maeva Delacrétaz, Aliki Vasilakou, Marta Monguió-Tortajada, Léa Bernaleau, Maxime Dubois, Vanja Sisirak, Manuele Rebsamen

**Affiliations:** ^a^ Department of Immunobiology, University of Lausanne, Epalinges 1066, Switzerland; ^b^ CNRS-UMR 5164, Immunoconcept, Bordeaux University, Bordeaux 33000, France

**Keywords:** autoimmunity, SLE, Toll-like receptors, SLC15A4, TASL

## Abstract

Nucleic acid sensing by endolysosomal Toll-like receptors is critically involved in systemic lupus erythematosus (SLE) and related autoimmune diseases. Downstream of TLR7, TLR8, and TLR9, the SLE-associated SLC15A4–TASL complex selectively mediates IRF5 activation, while being dispensable for NF-κB and MAPK pathways. Here, we show that the SLC15A4–TASL signaling axis is broadly required for disease development and pathogenesis across three complementary genetic SLE models, reflecting different etiologies. Genetic ablation of *Tasl* (*Tasl*^KO^) and its paralogue *Tasl2* (*Tasl*^DKO^) ameliorated or fully prevented autoimmune manifestations resulting from *Fas*^lpr^ loss of function, respectively. Furthermore, SLC15A4–TASL complex deficiency was sufficient to protect from disease, including splenomegaly, immune activation, and autoantibody formation, driven by the patient-derived *Tlr7*^Y264H^ gain-of-function mutation, even in the presence of functional NF-κB and MAPK responses. Last, *Tasl*^DKO^ splenocyte transfer into lymphopenic *Dnase1l3*-deficient mice demonstrated that protection extends to DNA-driven autoimmunity, in which TLR7 and TLR9 act redundantly, strongly supporting a B cell–intrinsic role. Notably, SLC15A4–TASL complex deficiency blunted the generation of pathogenic age-associated B cells in all three autoimmune models as well as in aged animals. Collectively, these data demonstrate the central role of the SLC15A4–TASL complex in SLE, supporting its potential as therapeutic target.

## Significance

Systemic lupus erythematosus is a severe, complex, and heterogeneous autoimmune disease in which aberrant activation of endolysosomal nucleic acids sensing Toll-like receptors plays a central pathogenic role. The SLC15A4–TASL complex selectively activates inflammatory IRF5 signaling downstream of these receptors and is genetically associated with human lupus. Using three complementary mouse models reflecting distinct causes of lupus-like disease, we show that disruption of the SLC15A4–TASL complex broadly prevents autoimmunity, including immune activation, pathogenic B cell responses, and autoantibody production. Protection was observed even when parallel inflammatory pathways remained intact. Our findings identify the SLC15A4–TASL signaling axis as a central and nonredundant driver of lupus pathogenesis and support the therapeutic targeting of this pathway in systemic lupus erythematosus and related autoimmune diseases.

Systemic lupus erythematosus (SLE) is a chronic autoimmune disease involving aberrant nucleic acid–driven responses contributing to breakdown of immunological self-tolerance (1). The complexity of SLE is reflected by the clinical heterogeneity observed across patients, with multiple immune cell populations shown to participate in disease pathogenesis affecting different organs (2–4). Similar to other rheumatic autoimmune diseases, SLE exhibits a striking sex bias with women accounting for up to 90% of affected individuals (5, 6). Beside the influence of sex hormones, X-linked gene dosage and dysregulated X chromosome inactivation (XCI) are recently emerging as central contributors to female-biased susceptibility (7, 8). Among the prominent features of SLE are the presence of autoantibodies and, in large portion of patients, a type I interferon (IFN-I) signature (9). In particular, age-associated B cells (ABC) in mice and IgD^−^ CD27^−^ double negative B cells (DN2) subsets in humans have been linked to disease development and severity by contributing to emergence of autoreactive B cells (10–12). Insights into these pathogenic mechanisms led to the development of targeted treatment strategies, including type I interferon signaling blockade and B cell depleting or modulating therapies (13–17). Notably, despite the complex environmental and polygenic components of SLE, multiple genetic and functional studies converge toward a critical role of the endolysosomal nucleic acid–sensing Toll-like receptor (TLR) pathway, particularly TLR7 (18–20). Indeed, dysregulated TLR7 signaling has emerged as a central driver of lupus pathogenesis in human and mouse (21–24). This has been further supported by the recent identification of monogenic, disease-causing variants in TLR7 and its regulator UNC93B1 in SLE patients (25, 26).

Investigating SLE-associated genes, we previously reported that the lysosomal solute carrier SLC15A4 forms a signaling complex with the immune adaptor TASL, which is essential for TLR7/9-induced IRF5 activation (27). Mechanistic and structural studies further clarified the SLC15A4–TASL binding mode and revealed the druggable nature of the complex, which is selectively involved in IRF5 responses, while dispensable for NF-κB and MAPK signaling (28–32). We recently showed that knock-out of *Tasl* and its murine paralogue *Tasl2* impaired TLR7/9 responses and antiviral immunity in vivo, phenocopying *Slc15a4-*deficient mice and confirming the critical role of this signaling axis (33). Notably, *Tasl*^KO^ conferred strong protection in pristane- and imiquimod-driven SLE-like models, in line with the human genetic association with the disease, suggesting a therapeutic potential for targeting this complex (33, 34). Moreover, *Tasl* is, like *Tlr7*, among the X-linked immune genes reported to facultatively escape X inactivation in both human and mouse, thus possibly contributing to the female sex bias (8, 35, 36). Given the diverse pathomechanisms contributing to SLE, it nevertheless remains unclear whether SLC15A4–TASL axis is broadly required for disease development beyond the described chemically inducible models. This represents also a critical question to evaluate the therapeutic potential of targeting this druggable complex (30, 37).

Here, we examine the role of the SLC15A4–TASL pathway in three different and complementary genetic SLE models driven by *Fas* loss of function (*Fas*^lpr^) (38, 39), a patient-derived TLR7^Y264H^ gain-of-function mutation (*Tlr7*^kik^ model) (40), or DNA-induced autoimmunity resulting from DNASE1L3 deficiency (41). Across these settings, we show that genetic disruption of the SLC15A4–TASL axis conferred remarkable protection from disease development, characterized by profound block in ABC accumulation, absence of pathogenic autoantibody production and downstream tissue pathology. Collectively, our findings demonstrate that selective impairment of the SLC15A4–TASL–IRF5 pathway is sufficient to prevent lupus-like disease manifestations, even in the presence of intact NF-κB and MAPK signaling, thus establishing this axis as a central and nonredundant mediator of nucleic acid–driven autoimmunity.

## Results

### TASL Deficiency Confers Protection in the *Fas*^lpr^ Lupus Model.

To assess the relevance of SLC15A4–TASL axis in SLE beyond chemically induced models, we investigated the contribution of this pathway in genetically driven disease models. We first selected the well-established MRL-*Fas*^lpr^ spontaneous lupus model on the C57BL/6J background (B6.MRL-Faslpr/J), in which defective FAS-mediated apoptosis leads to progressive lymphoproliferation and systemic autoimmunity (38, 39). Given prior evidence that *Slc15a4*-deficient *feeble* mice are largely protected in this model (42), and our earlier findings that *Tasl2* single deficiency does not affect early pristane-induced inflammatory responses (33), we focused our analysis on *Tasl* single knockout (*Tasl*^KO^) and *Tasl*/*Tasl2* double knockout (*Tasl*^DKO^) mice. Indeed, we further confirmed that single *Tasl2* deficiency did not impact pristane-induced disease progression by assessing autoantibody production 4 mo after pristane injection. *Tasl2*-deficient mice displayed antinuclear antibody (ANA) staining patterns and anti-Sm/RNP autoantibody levels comparable to wild-type (WT) controls (*SI Appendix*, Fig. S1 *A* and *B*), corroborating our earlier findings indicating no or minimal contribution of TASL2 in this disease context.

**Fig. 1.**
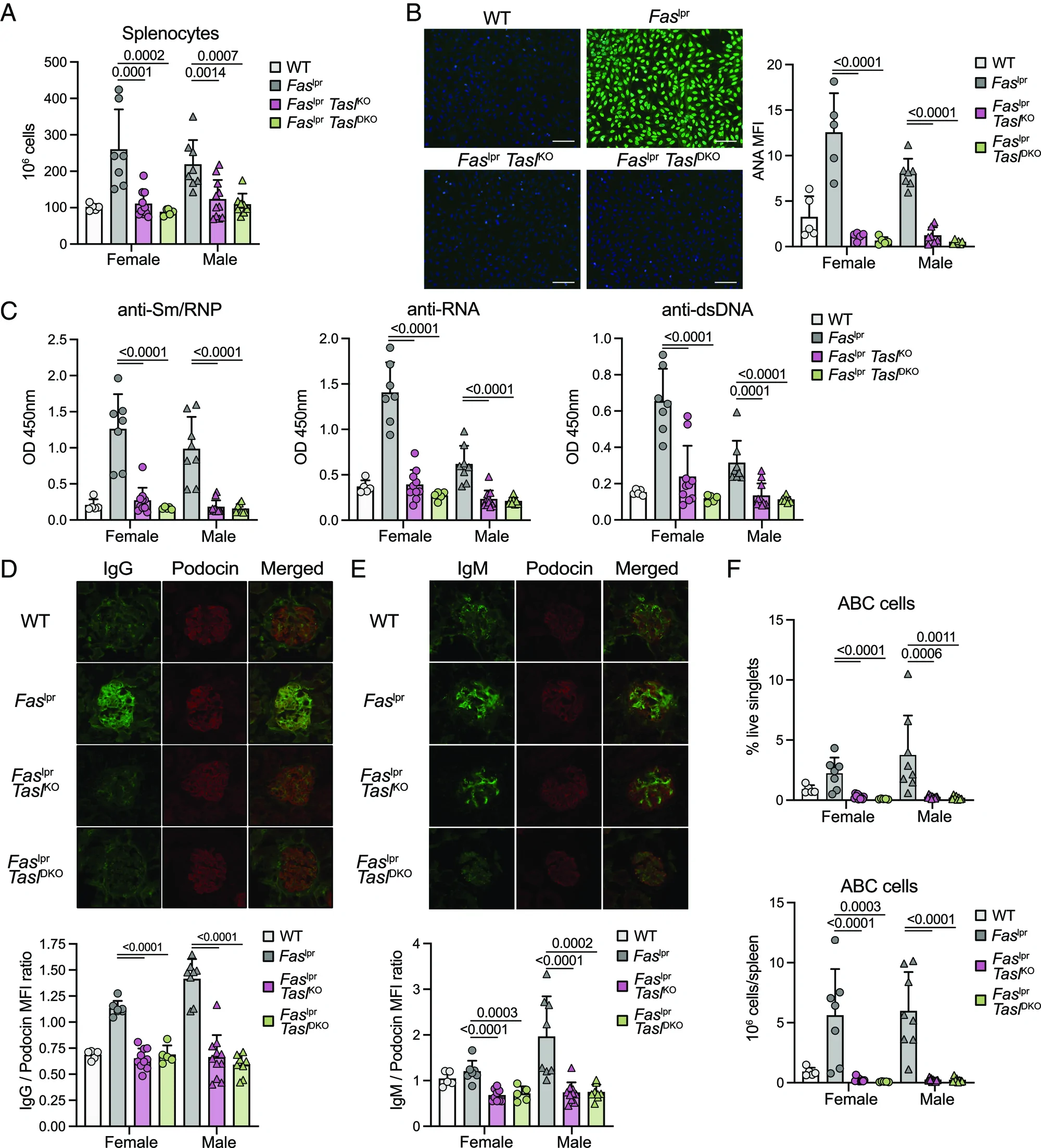
TASL deficiency protects from *Fas*^lpr^-induced autoimmunity. (*A*) Spleen cellularity at 8 mo of age. (*B*) Serum antinuclear antibodies (ANA, 1/300) assessed by Hep-2 immunofluorescence. Representative images and MFI quantification are shown. (Scale bar, 100 μm.) (*C*) Autoantibody levels against indicated self-antigens in sera of 8 mo old mice measured by ELISA. (*D* and *E*) IgG (*D*) and IgM (*E*) immunocomplex deposition in podocin-marked kidney glomeruli. Representative images and MFI ratio (Ig/podocin) are shown. (*F*) Percentage and total number of ABC cells (gated as CD19^+^ B220^+^ CD21^−^ CD23^−^ MHC-II^high^ CD11c^+^) in the spleen. Data in all graphs are shown as means ± SD. Statistical analysis was performed with one-way ANOVA using Dunnett’s multiple comparisons test to *Fas*^lpr^ mice. Circle datapoints represent females and triangles males. (*A* and *C–F*) *n* = 5 to 11, (*B*) *n* = 5 to 7 per genotype.

Next, we assessed *Fas*^lpr^ mice, and, consistent with expected disease progression, we observed pronounced splenomegaly by 8 mo of age. Strikingly, this was largely prevented in *Fas*^lpr^ *Tasl*^KO^ and fully normalized in *Fas*^lpr^ *Tasl*^DKO^ animals, both females and males (Fig. 1*A* and *SI Appendix*, Fig. S1*C*). While *Tasl* single deficiency was sufficient to confer near-complete protection, double deficiency further enhanced this effect. In contrast, despite a delayed onset, lymphadenopathy remained detectable in *Fas*^lpr^ *Tasl*^KO^ and *Tasl*^DKO^ mice at 8 mo, indicating that TASL/TASL2 loss does not prevent lymphoproliferation resulting from *Fas* loss of function (*SI Appendix*, Fig. S1*D*). Focusing on systemic manifestations of autoimmunity, ANA staining revealed a global impairment of autoantibody generation in *Fas*^lpr^ *Tasl*^KO^ and *Tasl*^DKO^, with virtually no nuclear and cytoplasmic reactivity compared to *Fas*^lpr^ controls (Fig. 1*B*). Accordingly, anti-Sm/RNP, anti-RNA, and anti-dsDNA autoantibody production was largely prevented upon *Tasl* deficiency, with *Fas*^lpr^ *Tasl*^DKO^ showing levels comparable to WT controls (Fig. 1*C*). *Fas*^lpr^ female mice exhibited higher autoantibody titers than males; nevertheless, *Tasl*^DKO^ normalized autoantibody levels in both sexes. Importantly, we further observed that IgG and IgM immune complex deposition in kidney glomeruli, a key pathogenic mechanism in lupus nephritis, was strongly reduced in *Fas*^lpr^ *Tasl*^KO^ and *Tasl*^DKO^ mice (Fig. 1 *D* and *E*).

Flow cytometry analysis of the splenic immune compartment in *Fas*^lpr^ mice showed the expected expansion of CD4^+^ T cells, alongside with the accumulation of double-negative (DN) T cells (CD3ε^+^ CD4^−^ CD8α^−^) and B220^+^ CD19^−^ B cell population, and consequential relative reduction of plasmacytoid dendritic cells (pDC, CD11c^+^ B220^+^ Siglec-H^+^) frequencies. These alterations were strongly reduced in *Fas*^lpr^ *Tasl*^KO^ and even more prominently in *Fas*^lpr^ *Tasl*^DKO^ mice, although DN T cells and B220^+^ CD19^−^ B cells remained detectable (*SI Appendix*, Fig. S1 *E–G*). Within the conventional B220^+^ CD19^+^ compartment, we did not observe major changes in the distribution of IgM and IgD subsets, with the exception of an increased IgM^−^ IgD^−^ population in *Fas*^lpr^ females which correlated with a decrease in single positive populations (*SI Appendix*, Fig. S1*H*). Notably, further analysis of the B cell compartment revealed an impairment of marginal zone (MZ) B cells (CD21^+^ CD23^low^) and a marked expansion of CD21^−^ CD23^−^ DN B cells in *Fas*^lpr^ mice, a subset that includes ABCs (*SI Appendix*, Fig. S1 *I* and *J*). Beyond BCR signaling and IL-21, ABCs differentiation critically depends on endosomal TLR activation (10, 11, 43). ABCs have been implicated in lupus pathogenesis, in particular for their enhanced antigen presenting functions leading to aberrant T cell activation and propensity to differentiate into (auto)antibody-secreting cells (12, 44–46). We therefore assessed ABCs (defined as CD11c^+^ MHC-II^high^) within DN (CD21^−^ CD23^−^) B cells. Remarkably, while ABCs accumulate in *Fas*^lpr^, this population was virtually absent in both *Fas*^lpr^ *Tasl*^KO^ and *Fas*^lpr^ *Tasl*^DKO^, with frequencies even lower than those observed in age-matched WT controls (Fig. 1*F* and *SI Appendix*, Fig. S1*K*). MZ B cell populations were similarly restored (*SI Appendix*, Fig. S1 *I* and *J*).

Collectively, these data demonstrate that the TASL-dependent pathway is essential for the development of lupus-like autoimmunity driven by *Fas*^lpr^ loss of function by controlling autoantibody production, immune complex deposition in glomeruli and, notably, the expansion of pathogenic ABCs.

### The SLC15A4–TASL Signaling Axis Is Critical in the SLE Patient-Derived *Tlr7^Y264H^* Gain-of-Function Mouse Model.

To determine whether the protective effect of SLC15A4–TASL complex disruption extends beyond FAS-dependent autoimmunity in settings closely reflecting human disease etiology, we next took advantage of the recently developed Tlr7-kika model, in which a knock-in gain-of-function mutation (Y264H) in *Tlr7* identified in a child with severe SLE drives spontaneous systemic autoimmunity (40). We therefore crossed Tlr7-kika (*Tlr7*^kik^) mice with *Tasl*^DKO^ or *Slc15a4*-deficient *feeble* (*Slc15a4*^F/F^) animals to achieve full block of SLC15A4–TASL signaling and assessed disease development.

**Fig. 2.**
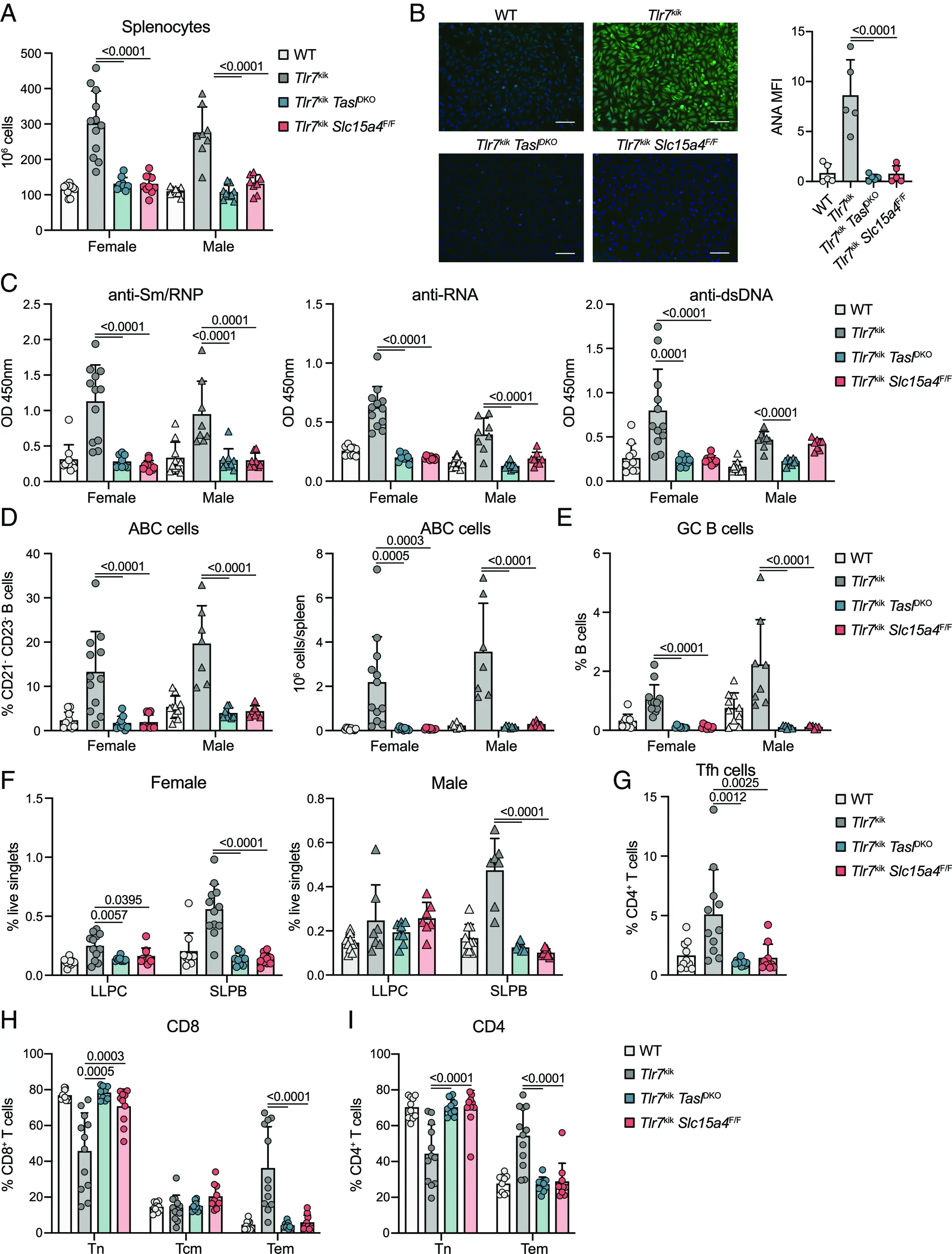
SLC15A4–TASL axis is crucial for SLE induction by *Tlr7*^kik^ gain of function. (*A*) Spleen cellularity at 12 wk of age. (*B*) Serum antinuclear antibodies (ANA, 1/200) assessed by Hep-2 immunofluorescence. Representative images and MFI quantification are shown. (Scale bar, 100 μm.) (*C*) Autoantibody levels against indicated self-antigens in sera of 12 wk old mice measured by ELISA. (*D–F*) Flow cytometry analysis of splenic B cells (CD19^+^ B220^+^) showing percentage of ABC cells (MHC-II^high^ CD11c^+^) in DN B cells (CD21^−^ CD23^−^) and total number per spleen (*D*), percentage of germinal center (GC) B cells (Fas^+^ GL7^+^) (*E*) and long lived plasma cells (LLPC, CD138^+^ B220^−^) and short-lived plasmablasts (SLPB, CD138^+^ B220^+^) (*F*). (*G–I*) Flow cytometry analysis of splenic T cells (CD3ε^+^) showing percentage of T follicular helper (Tfh, CXCR5^+^ PD1^+^) cells from CD4^+^ T cells (*G*), percentage of naïve (Tn, CD62L^+^ CD44^−/low^), central memory (Tcm, CD62L^+^ CD44^+^), and effector memory (Tem, CD62L^−^ CD44^+^) within CD8^+^ T cell population (*H*) and percentage of naïve (Tn) and effector memory (Tem) in CD4^+^ T cell population (*I*). Statistical analysis was performed with one-way ANOVA using Tukey’s multiple comparisons test, means ± SD. Circle datapoints represent females and triangles males. (*A* and *C–I*) *n* = 8 to 12 per genotype, (*B*) *n* = 5 females per genotype.

Consistent with the initial report, *Tlr7*^kik^ mice developed marked splenomegaly at the age of 12 wk, reflecting progressive immune cell proliferation. Strikingly, splenic enlargement was completely prevented in both *Tlr7*^kik^ *Tasl*^DKO^ and *Tlr7*^kik^ *Slc15a4*^F/F^ mice, with spleen sizes indistinguishable from WT controls (Fig. 2*A*). This was further supported by normalization of immune cell composition, including correction of the expanded B cell compartment (*SI Appendix*, Fig. S2*A*).

Monitoring autoantibody by ANA staining revealed robust nuclear and cytoplasmic reactivity in *Tlr7*^kik^, whereas sera from *Tlr7*^kik^ *Tasl*^DKO^ and *Tlr7*^kik^ *Slc15a4*^F/F^ animals were negative, like WT controls. This phenotype was similar in both females and males (Fig. 2*B* and *SI Appendix*, Fig. S2*B*). Consistently, we observed that *Tasl*^DKO^ and *Slc15a4*^F/F^ normalized anti-Sm/RNP, anti-RNA, and anti-dsDNA autoantibodies in *Tlr7*^kik^ mice to background WT levels (Fig. 2*C*).

Examining the B cell compartment, we detected a strong reduction of MZ B cells in *Tlr7*^kik^, which was rescued by *Tasl*^DKO^ and *Slc15a4*^F/F^, while the overall frequency of CD21^−^ CD23^−^ DN B cells was comparable across genotypes, with a modest tendency toward increased abundance in *Tlr7*^kik^ mice (*SI Appendix*, Fig. S2*C*). In contrast, CD11c^+^ MHC-II^high^ ABCs were readily detectable in *Tlr7*^kik^ mice but virtually absent in *Tlr7*^kik^ *Tasl*^DKO^ and *Tlr7*^kik^ *Slc15a4*^F/F^, mirroring the protective effect observed in *Fas*^lpr^ mice (Fig. 2*D* and *SI Appendix*, Fig. S2*D*). This demonstrates the strict requirement of the SLC15A4–TASL axis for ABC generation and accumulation downstream of TLR7 signaling.

Despite the extrafollicular origin of pathogenic B cells in this model, *Tlr7*^kik^ mice show prominent spontaneous germinal-centers formation during disease development (40). Both GC B cell formation and short-lived plasmablasts (SLPB) expansion observed in *Tlr7*^kik^ were abrogated in *Tlr7*^kik^ *Tasl*^DKO^ and *Tlr7*^kik^ *Slc15a4*^F/F^ mice (Fig. 2 *E* and *F* and *SI Appendix*, Fig. S2 *E* and *F*). Consistent with this, the elevated proportion of follicular helper T (Tfh) cells in *Tlr7*^kik^ mice was normalized (Fig. 2*G* and *SI Appendix*, Fig. S2*G*). Furthermore, we assessed the T cell activation phenotypes which are driven by cell-extrinsic cues, likely due to a chronic inflammatory environment (40). CD8^+^ T cells from *Tlr7*^kik^ mice displayed a pronounced effector memory phenotype, characterized by increased CD44^high^ CD62L^−^ cells at the expense of naïve CD44^low^ CD62L^+^ T cells, while CD44^high^ CD62L^+^ central memory populations remained largely unchanged (Fig. 2*H* and *SI Appendix*, Fig. S2*H*). A similar skewing toward effector memory differentiation was observed within the CD4^+^ T cell compartment (Fig. 2*I* and *SI Appendix*, Fig. S2*I*). Notably, these T cell activation phenotypes were normalized in *Tlr7*^kik^ *Tasl*^DKO^ and *Tlr7*^kik^ *Slc15a4*^F/F^ mice to steady-state WT control levels. Last, analysis of splenic pDCs in *Tlr7*^kik^ mice revealed the expected reduced frequency and the activated phenotype, as assessed by downregulation of Siglec-H and upregulation of CD86 and MHC-II (*SI Appendix*, Fig. S3 *A–D*). *Tlr7*^kik^ *Tasl*^DKO^ and *Tlr7*^kik^ Slc15a4^F/F^ mice showed higher pDC frequency and normalized CD86 level. Interestingly, downregulation of Siglec-H and upregulation of MHC-II was only partially rescued, pointing to a possible involvement of TLR7-driven but SLC15A4–TASL complex independent responses via the NF-κB and/or MAPK pathways.

Together, these data demonstrate that the SLC15A4–TASL complex is essential for TLR7 gain-of-function-induced lupus-like pathogenesis, as its loss prevents ABC accumulation, spontaneous GC responses, autoantibody production, and secondary T cell activation.

### *Tasl* Loss, But Not *Tasl2*^KO^ or *Slc15a4* Haploinsufficiency, Is Sufficient to Prevent Disease in *Tlr7*^kik^ Mice.

Previous studies showed that haploinsufficiency of IRF5 significantly attenuates disease in MRL/lpr and *FcγRIIB^−/−^Yaa* lupus models (47, 48). Similarly, SLC15A4 heterozygous loss prevents disease manifestation in BXSB and NZB/W F1 lupus models (49, 50). We therefore investigated whether single deficiency of *Tasl* or *Tasl2*, or heterozygous loss of *Slc15a4* (*Slc15a4*^F/WT^) would be sufficient to limit autoimmunity driven by constitutive *Tlr7*^kik^ gain of function, or whether full abrogation of the complex is required for disease protection in this patient-derived model.

**Fig. 3.**
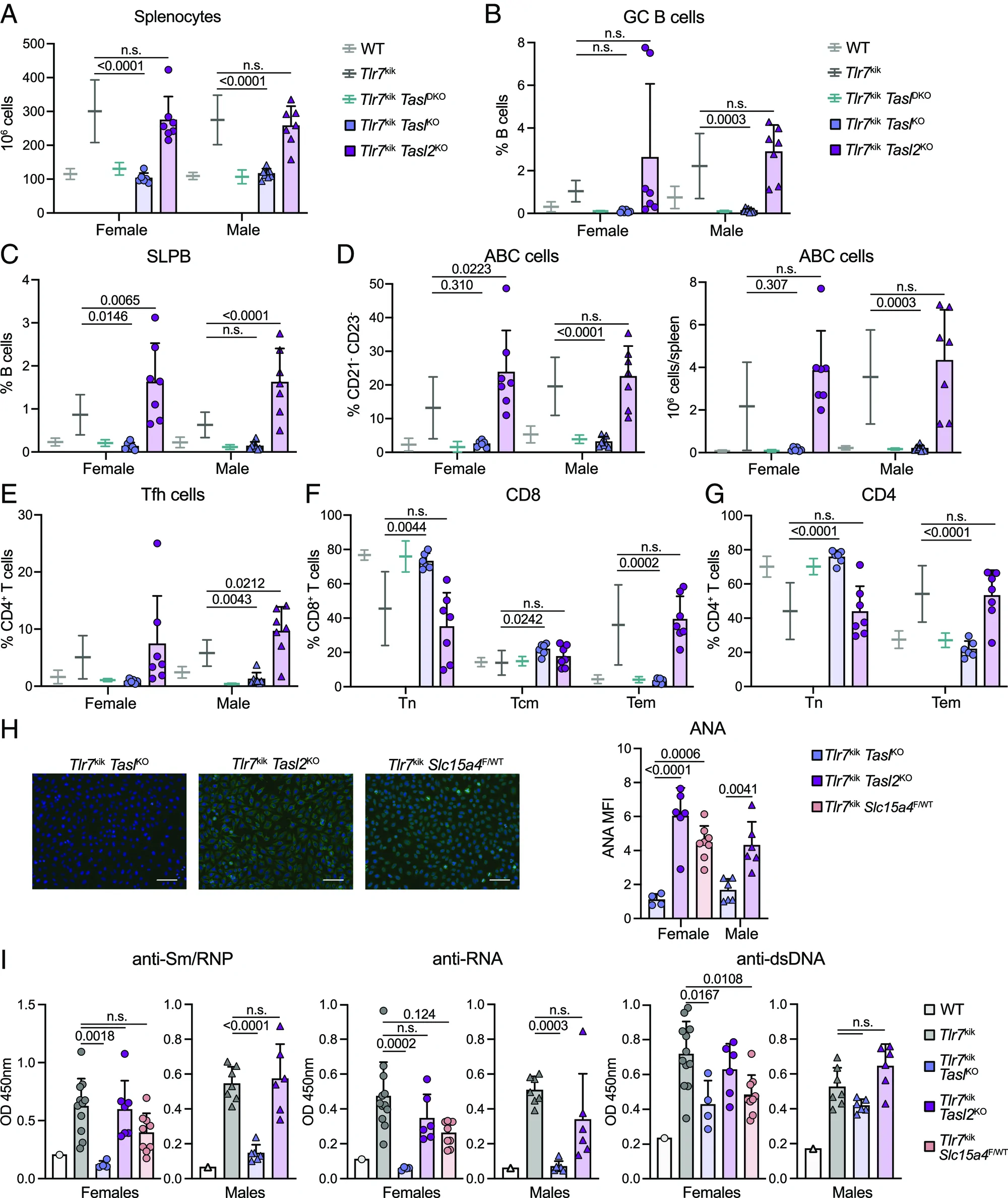
Single *Tasl*^KO^, but not *Tasl2*^KO^ nor *Slc15a4*^F/WT^, confers protection to *Tlr7*^kik^. (*A*) Spleen cellularity at 12 wk of age. (*B–D*) Flow cytometry analysis of splenic B cells (CD19^+^ B220^+^) showing percentage of GC B cells (Fas^+^ GL7^+^) (*B*), SLPBs (B220^+^ CD138^+^) (*C*) and percentage of ABC cells (MHC-II^high^ CD11c^+^) in DN B cells (CD21^−^ CD23^−^) and total number per spleen (*D*). (*E–G*) Flow cytometry analysis of splenic T cells (CD3ε^+^) showing percentage of T follicular helper (Tfh, CXCR5^+^ PD1^+^) cells from CD4^+^ T cells (*E*), percentage of naïve (Tn, CD62L^+^ CD44^−/low^), central memory (Tcm, CD62L^+^ CD44^+^), and effector memory (Tem, CD62L^−^ CD44^+^) within CD8^+^ T cell population (*F*) and percentage of naïve (Tn) and effector memory (Tem) in CD4^+^ T cell population (*G*). (*H*) Serum antinuclear antibodies (ANA, 1/200) assessed by Hep-2 immunofluorescence. Representative images and MFI quantification are shown. (Scale bar, 100 μm.) (*I*) Autoantibody levels against indicated self-antigens in sera of 12 wk old mice measured by ELISA. For direct comparison, *Tlr7*^kik^ sera used in Fig. 2*C* were rerun together with *Tlr7*^kik^ *Tasl*^KO^, *Tlr7*^kik^ *Tasl2*^KO^, and *Tlr7*^kik^ *Slc15a4*^F/WT^ samples. In panels *A*–*G*, data on WT, *Tlr7*^kik^, and *Tlr7*^kik^ *Tasl*^DKO^ mice from Fig. 2 are shown as line representing mean with ±SD to facilitate comparison with *Tlr7*^kik^ *Tasl*^KO^ and *Tlr7*^kik^ *Tasl2*^KO^. Statistical analysis was performed with one-way ANOVA using Tukey’s multiple comparisons test, means ± SD. Circle datapoints represent females and triangles males. (*A–G*) *n* = 6 to 8, (*H*) *n* = 4 to 8, (*I*) *n* = 4 to 11 per genotype.

Consistent with our previous observation of *Tasl*^KO^ having a dominant role compared to *Tasl2*^KO^ [*SI Appendix*, Fig. S1 *A* and *B*; and (33)], spleen size and cellularity were normalized in *Tlr7*^kik^ *Tasl*^KO^, whereas *Tasl2*^KO^ or *Slc15a4* heterozygosity failed to correct splenomegaly (Fig. 3*A* and *SI Appendix*, Fig. S4*A*). Within the B cell compartment, spontaneous GC B cell formation and SLPB expansion were abolished in *Tlr7*^kik^ *Tasl*-deficient animals but remained elevated in *Tlr7*^kik^ *Tasl2*^KO^ and *Tlr7*^kik^ *Slc15a4*^F/WT^ mice (Fig. 3 *B* and *C* and *SI Appendix*, Fig. S4 *B* and *C*). Furthermore, analysis of ABC cells revealed that both the frequency in DN B cells and the total splenic numbers were abrogated in *Tlr7*^kik^ *Tasl*^KO^ animals, whereas *Tasl2* deficiency or *Slc15a4* haploinsufficiency had no substantial effect (Fig. 3*D* and *SI Appendix*, Fig. S4*D*). Consistently, the elevated proportion of Tfh cells observed in *Tlr7*^kik^ mice was normalized in *Tlr7*^kik^ *Tasl*^KO^ but persisted in *Tlr7*^kik^ *Tasl2*^KO^ and *Tlr7*^kik^ *Slc15a4*^F/WT^ mice (Fig. 3*E* and *SI Appendix*, Fig. S4*E*). Moreover, the skewing of CD4^+^ and CD8^+^ T cells toward activated effector memory phenotype at the expense of naïve cells was fully corrected in *Tlr7*^kik^ *Tasl*^KO^, while remaining unchanged in *Tlr7*^kik^ *Tasl2*^KO^ and *Tlr7*^kik^ *Slc15a4*^F/WT^ mice (Fig. 3 *F* and *G* and *SI Appendix*, Fig. S4 *F–I*). Last, pDC frequency was restored and CD86 expression normalized in *Tlr7*^kik^ *Tasl*^KO^, similar as *Tlr7*^kik^ *Tasl*^DKO^, while the phenotype in *Tlr7*^kik^ *Tasl2*^KO^ was comparable to *Tlr7*^kik^ mice (*SI Appendix*, Fig. S4 *J* and *K*).

ANA assessment revealed markedly reduced staining in *Tlr7*^kik^ *Tasl*^KO^ serum, whereas *Tlr7*^kik^ *Tasl2*^KO^ and *Tlr7*^kik^ *Slc15a4*^F/WT^ serum still exhibited relatively strong reactivity (Fig. 3*H*). Moreover, anti-Sm/RNP and anti-RNA autoantibodies were normalized in *Tlr7*^kik^ *Tasl*^KO^ but remained elevated in *Tlr7*^kik^ *Tasl2*-deficient and *Tlr7*^kik^ *Slc15a4* heterozygous animals (Fig. 3*I*). Reduction in anti-dsDNA in *Tlr7*^kik^ *Tasl*^KO^ mice was milder possibly indicating contribution of *Tasl2* in DNA-specific autoreactivity (Fig. 3*I*).

Together, these data demonstrate that loss of TASL, but not of TASL2, is sufficient to prevent TLR7-driven immune activation and autoantibody production. This further supports the dominant role of TASL previously observed, likely reflecting different expression pattern of these two paralogues across immune cell types (33). Interestingly, partial reduction of signaling output in *Slc15a4*^F/WT^ was not sufficient to confer protection, indicating the need of a profound impairment of the SLC15A4–TASL axis to prevent TLR7 gain-of-function-driven disease.

**Fig. 4.**
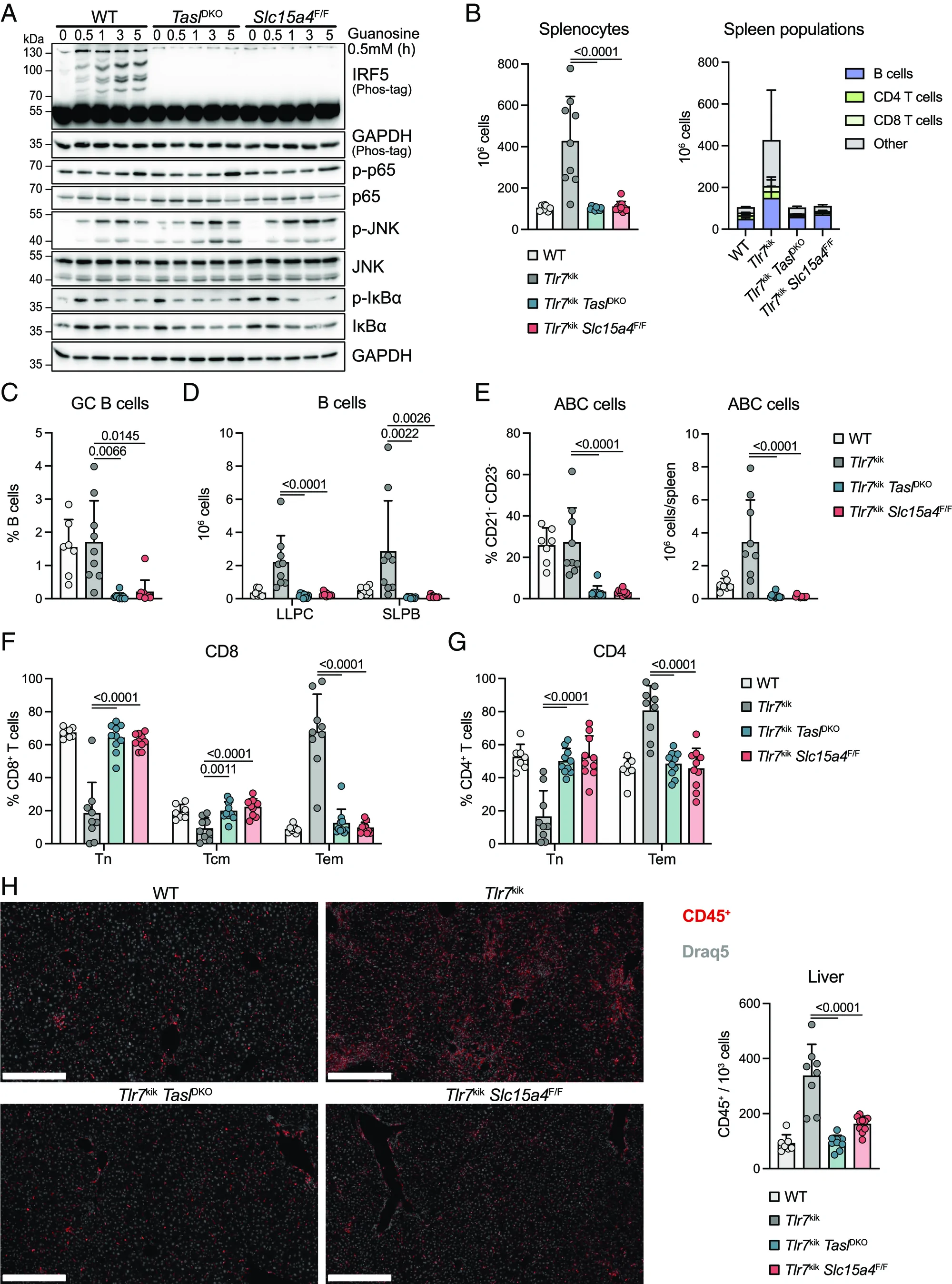
Loss of SLC15A4–TASL complex prevents and not only delays disease development in *Tlr7*^kik^ mice. (*A*) WT, *Tasl*^DKO^, and *Slc15a4*^F/F^ splenic B cells were stimulated with guanosine (500 μM) and analyzed by immunoblotting. Data representative of two independent experiments. (*B*) Spleen cellularity and mean proportion of main immune cell types in 22 to 23 wk old mice. (*C–E*) Flow cytometry analysis of splenic B cells (CD19^+^ B220^+^) showing percentage of GC B cells (Fas^+^ GL7^+^) (*C*), total number of long-lived plasma cells (LLPC, CD138^+^ B220^−^) and short-lived plasmablasts (SLPB, CD138^+^ B220^+^) (*D*) and percentage of ABC cells (MHC-II^high^ CD11c^+^) in DN B cells (CD21^−^ CD23^−^) and total number per spleen (*E*). (*F* and *G*) Flow cytometry analysis of splenic T cells (CD3ε^+^) showing percentage of naïve (Tn, CD62L^+^ CD44^−/low^), central memory (Tcm, CD62L^+^ CD44^+^), and effector memory (Tem, CD62L^−^ CD44^+^) cells in CD8^+^ T cell population (*F*) and percentage of naïve (Tn) and effector memory (Tem) cells within CD4^+^ T cell population (*G*). (*H*) Representative microscopy images of liver sections for each genotype with anti-CD45 staining and Draq5 as counterstain of nuclei with quantification. (Scale bar, 250 μm.) Statistical analysis was performed with one-way ANOVA using Tukey’s multiple comparisons test, means ± SD. (*B–H*) *n* = 7 to 10 per genotype.

SLC15A4–TASL complex deficiency selectively impairs IRF5 activation while leaving NF-κB and MAPK responses unaffected (33, 34), which could, in principle, sustain inflammatory pathology and disease development over time. While our data showing that *Tlr7*^kik^ *Tasl*^DKO^ and *Tlr7*^kik^ *Slc15a4*^F/F^ mice are fully protected from autoimmune manifestations at 12 wk of age suggest that *Tlr7*^kik^-induced NF-κB and MAPK signaling is not sufficient to induce pathogenesis in the absence of IRF5 activation, we further assessed if SLC15A4–TASL loss fully prevented or only delayed disease development. The *Tlr7*^kik^ mutation has been shown to increase receptor sensitivity to guanosine, while this potentiation was not observed for synthetic agonist R848 (40). Therefore, we first wanted to confirm that SLC15A4–TASL complex deficiency selectively ablates IRF5, but not NF-κB and MAPK signaling upon guanosine stimulation, as we and others previously demonstrated for R848 (33, 34). To avoid possible confounding effects of an altered splenic compartment in *Tlr7*^kik^ compared to *Tlr7*^kik^ *Tasl*^DKO^ and *Tlr7*^kik^ *Slc15a4*^F/F^ mice, we assessed primary B cells from WT, *Tasl*^DKO^, and *Slc15a4*^F/F^ mice stimulated with guanosine. Whereas WT B cells displayed robust IRF5 activation under these conditions, IRF5 activation was abrogated in *Tasl*^DKO^ and *Slc15a4*^F/F^ cells (Fig. 4*A*). In contrast, NF-κB and MAPK pathway activation remained unaffected. Similar results were obtained with bone marrow–derived macrophages (*SI Appendix*, Fig. S5*A*).

Therefore, we next assessed disease progression at 22 to 23 wk of age. As expected, aged *Tlr7*^kik^ mice exhibited progressive immune cell proliferation and sustained expansion of activated immune compartments. In contrast, *Tlr7*^kik^ *Tasl*^DKO^ and *Tlr7*^kik^ *Slc15a4*^F/F^ mice remained protected, with spleen size and immune cell distributions comparable to age-matched WT controls (Fig. 4*B* and *SI Appendix*, Fig. S5*B*). Consistently, GC B cells, long-lived plasma cells, SLPBs, and ABC cells accumulation persisted in aged *Tlr7*^kik^ mice but remained markedly reduced in *Tlr7*^kik^ *Tasl*^DKO^ and *Tlr7*^kik^ *Slc15a4*^F/F^ animals (Fig. 4 *C*–*E* and *SI Appendix*, Fig. S5*C*). Likewise, the T cells activation phenotype present in *Tlr7*^kik^ mice was normalized in *Tlr7*^kik^ *Tasl*^DKO^ and *Slc15a4*^F/F^ (Fig. 4 *F* and *G*).

Reflecting the complete protection from *Tlr7*^kik^-induced immune activation and disease manifestations, *Tasl*^DKO^ and *Slc15a4*^F/F^ prevented immune infiltration in periphery, which contributes to tissue damage and end-organ pathology. Indeed, immunofluorescence analysis of CD45^+^ resident and infiltrating immune cells in the liver demonstrated that the increase observed in **Tlr7*^kik^* was absent in *Tlr7*^kik^ *Tasl*^DKO^ and *Slc15a4*^F/F^, which appeared as WT controls (Fig. 4*H*).

Collectively, these findings demonstrate that selective disruption of the SLC15A4–TASL complex prevents disease driven by *Tlr7* gain of function, highlighting the essential, nonredundant role of the IRF5-activating signaling branch even in presence of unaltered NF-κB and MAPK signaling.

### Lymphocyte-Specific *Tasl*^DKO^ Protects from DNA-Driven Autoimmunity Induced by DNASE1L3 Deficiency.

**Fig. 5.**
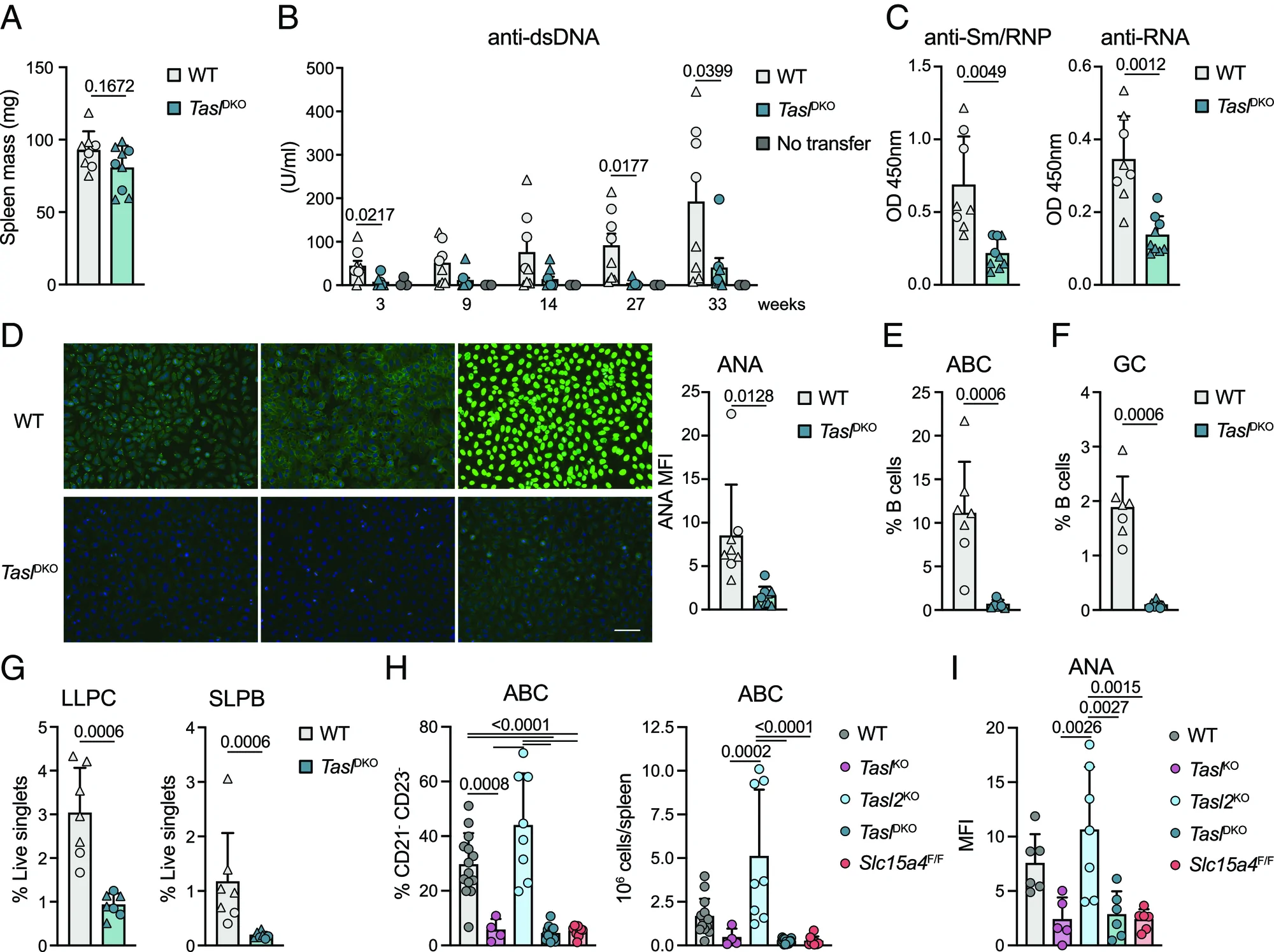
Lymphocyte-intrinsic *Tasl*^DKO^ protects from DNA-induced autoimmunity in DNASE1L3-deficient mice. (*A*) Spleen mass in *Dnase1l3*-*Rag1*^DKO^ mice reconstituted with WT or *Tasl*^DKO^ splenocytes analyzed 7 mo after adoptive transfer. (*B*) Longitudinal analysis of anti-dsDNA autoantibody levels in sera of *Dnase1l3*-*Rag1*^DKO^ reconstituted mice. (*C*) Autoantibody levels against indicated self-antigens in sera of *Dnase1l3*-*Rag1*^DKO^ mice 7 mo after adoptive transfer measured by ELISA. (*D*) Serum antinuclear antibodies (ANA, 1/80) assessed by Hep-2 immunofluorescence. Representative images and MFI quantification are shown. (Scale bar, 100 μm.) (*E–G*) Flow cytometry analysis of *Dnase1l3*-*Rag1*^DKO^ splenic B cells (CD19^+^ B220^+^) showing percentage of CD21^−^ CD11c^+^ ABC cells (*E*) and GC B cells (Fas^+^ GL7^+^) (*F*); and percentage of plasma cells (PC, CD138^+^ B220^−^) or plasmablasts (PB, CD138^+^ B220^+^) in live singlets (*G*). (*H*) Flow cytometry analysis of splenic B cells (CD19^+^ B220^+^) from 1-y-old female mice showing percentage of ABC cells (MHC-II^high^ CD11c^+^) in DN B cells (CD21^−^ CD23^−^) and total number per spleen. (*I*) Serum antinuclear antibodies (ANA, 1/50) in 1 y old female mice of indicated genotypes assessed by Hep-2 immunofluorescence. MFI quantification is shown. Circle datapoints represent female and triangles male *Dnase1l3*-*Rag1*^DKO^ recipient mice (*A–G*). Statistical analysis was performed with the Mann–Whitney test (*A* and *C–G*), two-way ANOVA with Šidák’s multiple comparisons test (*B*), and one-way ANOVA using Tukey’s multiple comparisons test (*H* and *I*), means ± SD. (*A–D*) *n* = 8 to 9, (*E–G*) *n* = 7, (*H*) *n* = 4 to 16, (*I*) *n* = 5 to 7 per genotype.

The results obtained in *Fas*^lpr^ and *Tlr7*^kik^ models raise the question of how broad the requirement of the SLC15A4–TASL axis is across diverse lupus etiologies. We therefore extended our investigations to DNA-driven autoimmunity, focusing on impaired DNA clearance resulting from reduced DNASE1L3 activity, which has been reported in SLE patients due to loss-of-function mutations or production of anti-DNASE1L3 autoantibodies (41, 51–53). The endonuclease DNASE1L3 is secreted by mononuclear phagocytes and mediates degradation of extracellular chromatin from apoptotic cells. Impaired activity leads to anti-dsDNA autoantibody production and systemic autoimmunity in both human and mouse (41, 51). Importantly, in *Dnase1l3^−/−^*-induced autoimmunity, TLR7 and TLR9 play largely redundant roles in driving pathogenesis, as demonstrated by observation that double *Tlr7^−/−^ Tlr9^−/−^* knockout is required to confer protection (54). DNASE1L3 has also been reported to exert a cell-extrinsic role in preventing the development of SLE. Although DNASE1L3 deficiency on a *Rag1*^KO^ background does not lead to autoimmunity, the transfer of WT splenocytes into *Dnase1l3-Rag1*^DKO^ mice induces autoreactivity (55). Therefore, we reconstituted *Dnase1l3-Rag1*^DKO^ mice with either WT or *Tasl*^DKO^ donor cells to evaluate the impact on a DNA-initiated model of SLE. Given that TASL is predominantly expressed in B cells, this approach allowed us to investigate whether a B cell–intrinsic defect in the SLC15A4–TASL axis is sufficient to confer protection, also considering the strong effects on ABC cells that we observed in the other models. We thus monitored these mice over 7 mo. Longitudinal analysis of reconstitution efficiency in the blood and, at the terminal point, in the spleen revealed no significant differences in total T and B cell frequencies as well as unaltered recipient’s monocyte and granulocyte populations (*SI Appendix*, Fig. S6 *A–E*). Consistently, spleen mass and cellularity were comparable between recipients of WT and *Tasl*^DKO^ splenocytes, with WT showing only a trend to increase likely reflecting ongoing autoimmunity (Fig. 5*A* and *SI Appendix*, Fig. S6*A*). Indeed, as expected we observed through longitudinal serological analysis a progressive increase in anti-dsDNA autoantibody titers in mice reconstituted with WT splenocytes. Notably, recipients of *Tasl*^DKO^ cells were protected, failing to mount comparable responses (Fig. 5*B*). Moreover, *Tasl* double deficiency significantly reduced also anti-Sm/RNP and anti-RNA autoantibody levels (Fig. 5*C*). In line with these findings, ANA staining revealed detectable autoreactivity in mice receiving WT, but not *Tasl*^DKO^ splenocytes (Fig. 5*D*).

Examining splenic B cell activation states, we observed accumulation of ABCs in mice reconstituted with WT splenocytes, whereas this population was blunted upon *Tasl*^DKO^ splenocyte transfer (Fig. 5*E*). Accordingly, in WT-reconstituted animals we detected spontaneous GC B cells as well as plasma cells and plasmablasts accumulation, while this was strongly reduced in *Tasl*^DKO^ receiving mice (Fig. 5 *F* and *G* and *SI Appendix*, Fig. S6*F*). Altogether, these data extend the protective effect of TASL deficiency to the context of defective DNA clearance and indicate that B cell-intrinsic, selective loss of this IRF5-activating axis is sufficient to prevent autoreactive humoral immunity even in the presence of an unaltered *Dnase1l3*^KO^ myeloid compartment.

Our data indicate that across three complementary SLE models, disruption of the SLC15A4–TASL axis blunted autoimmune ABC populations. Notably, in both the *Fas*^lpr^ and *Tlr7*^kik^ models, SLC15A4 and TASL deficiency not only prevented pathological ABCs expansion but reduced ABC frequencies to levels below those detected in WT controls (Figs. 1*F* and 4*E*). We therefore assessed whether TASL dependency extends to ABCs development during physiological aging. Analysis of 1-y-old mice revealed that, while WT animals accumulate ABCs as expected, this population was virtually ablated in *Tasl*^KO^, *Tasl*^DKO^, and *Slc15a4*^F/F^ mice, with *Tasl2*^KO^ having no effect (Fig. 5*H* and *SI Appendix*, Fig. S6*G*). Consistent with this, ANA autoreactivity in aged mice was diminished in absence of functional TASL signaling (Fig. 5*I*).

## Discussion

The pathogenesis of SLE is complex, with monogenic and nonmonogenic forms resulting from multifactorial etiologies, leading to diverse clinical manifestations (1, 4). This intrinsic heterogeneity represents a challenge to identify broadly effective therapeutic strategies (1, 4, 17).

In this study, we identified the SLC15A4–TASL signaling axis as a nonredundant and critical mediator of autoimmunity across three mechanistically distinct genetic mouse models of lupus. These cover different upstream drivers of disease, including defective apoptosis and lymphoproliferation in *Fas*^lpr^ mice, increased sensitivity to nucleic acid ligands in *Tlr7*^kik^ animals, and impaired degradation of extracellular chromatin in *Dnase1l3* deficiency. Genetic disruption of this pathway conferred robust and durable protection from disease manifestations, including autoantibody production and the accumulation of ABC. These findings establish that the SLC15A4–TASL complex represents a key signaling node required for the development of systemic autoimmunity, supporting its validity as therapeutic target in SLE across different etiologies.

Disease symptoms in *Fas*^lpr^ mice were either largely alleviated or fully blocked by TASL single- and double deficiency, respectively. While we observed rescue in SLE-like phenotypes, the primary *Fas*-deficiency-driven lymphoproliferation, manifested as lymphadenopathy, was still present. This contrasts with normalized spleen size in TASL KOs, where splenomegaly in *Fas*^lpr^ is largely a secondary effect of emerging autoimmunity. Although the *Fas*^lpr^ model is historically widely used and recapitulates key features of autoimmune SLE-like symptoms, loss-of-function mutations in *Fas* are generally not associated with SLE in humans but instead lead to autoimmune lymphoproliferative syndrome (ALPS) (56). To more directly address disease mechanisms relevant to human SLE, we extended our analysis to animal models representing human patient-derived *Tlr7* gain of function (*Tlr7*^kik^) and *Dnase1l3* deficiency, both of which reflect initiation and progression of clinically relevant pathogenic processes. In recent years, multiple studies have identified monogenic gain-of-function mutations in the *TLR7* or *UNC93B1* genes that cause SLE in patients (25, 26). The first TLR7 mutation (Y264H) described in the patient recapitulate disease development in knock-in *Tlr7^Y264H^* kika mouse, which display a rapid and severe disease progression (40). This phenotype arises from enhanced TLR7 signaling driven by increased sensitivity of TLR7^Y264H^ for the pocket 1 ligand guanosine (40). Moreover, a recent study described that TLR7^Y264H^ mutation also reduced binding to inhibitory 2′-O-methylated guanosine RNA species, thereby contributing to chronic receptor activation (57). Despite the fact that constitutive TLR7 activation leads to severe disease manifestations, disruption of the SLC15A4–TASL complex in *Tasl*^DKO^ and *Slc15a4*^F/F^ conferred complete protection, which persisted even in aged animals, showing that complex-dependent signaling is essential for disease initiation. Notably, heterozygous loss of SLC15A4, previously reported to attenuate disease in other lupus models (49, 50), was insufficient to confer protection in the *Tlr7*^kik^ context. In contrast, single *Tasl*^KO^ was sufficient to abrogate disease, despite having residual IRF5 activity mediated by TASL2 in B cell compartment, while *Tasl2*^KO^ showed no effect on disease progression, consistent with our observation that *Tasl2*^KO^ provided no protection in pristane-induced autoimmunity (33). While this paralogue-specific effect is not relevant for human SLE patients as TASL2 is absent in human, these data raise nevertheless the question whether the different impact of TASL and TASL2 on TLR7 output is only quantitative or also qualitative.

Despite constitutive engagement of TLR7 signaling and the resulting severity of disease in the *Tlr7*^kik^ model, selective disruption of the SLC15A4–TASL–IRF5 axis is sufficient to abrogate disease, indicating that aberrant NF-κB and MAPK pathway activation is insufficient to break tolerance and trigger pathogenic humoral responses in vivo. Although a limitation of our study is the lack of direct comparison with IRF5-deficient *Tlr7*^kik^ mice to exclude the contribution of other potential downstream effector mechanisms to the full disease protection observed, these data are consistent with previous studies on *Irf5*^−/−^ mice and strongly support IRF5-dependent responses as the dominant driver of pathogenesis in nucleic acid–mediated autoimmunity (58–60).

Besides gain- and loss-of-function mutations directly affecting signaling components and negative regulators of nucleic-acid sensing pathways, another class of genes associated with monogenic SLE in human is represented by loss of function of DNA and RNA nucleases (61, 62). Among these, *DNASE1L3* is one of the strongest genetic risk factors associated with human SLE (41, 63). Moreover, recent reports identified neutralizing autoantibodies against DNASE1L3 inhibiting its enzymatic function in a significant proportion of patients with severe sporadic SLE, revealing impairment of DNASE1L3 activity as a common nongenetic mechanism promoting autoimmunity (52, 53).

While initiated by impaired DNA degradation, combined TLR7 and TLR9 deficiency is required to fully rescue disease phenotypes in DNASE1L3-deficient mice, with single deletion of TLR7 or TLR9 having only modest effects (54). Consistently, adoptive transfer of WT splenocytes into *Dnasel13*-*Rag*^DKO^ recipients induced DNA autoreactivity, whereas this did not occurr with *MyD88*- or *Unc93b1*-deficient splenocytes (55). Our finding that transfer of *Tasl*^DKO^ splenocytes into *Dnasel13*-*Rag^D^*^KO^ did not cause the development of autoreactivity demonstrates that SLC15A4–TASL complex deficiency effectively disrupts both TLR7- and TLR9-dependent pathogenic signaling, positioning the complex as a broader and potentially more effective therapeutic target than selected inhibition of TLR7/8, which already showed promising results in clinical trials (64–69). Similar to *Dnasel13*^KO^, a pathogenic role for TLR9 has been reported in PLD4- and TNIP1-deficient mouse lupus models (70–73). Of note, while *Tlr9* knockout exacerbates pathological manifestations in other mouse models, combined *Tlr7* deficiency resulted in full protection from disease, suggesting that SLC15A4–TASL inhibition should remain an effective strategy even in these settings (74, 75). Importantly, the adoptive transfer approach we used preserves intact nucleic acid–sensing responses, including type I interferon production and signaling, in the recipient’s innate compartment, indicating that lymphocyte-intrinsic *Tasl*^DKO^ deficiency is sufficient to prevent disease development even in a proinflammatory environment. These findings are consistent with previous studies demonstrating that B cell–intrinsic deletion of MyD88 or IRF5 is sufficient to attenuate disease in lupus models including *Fas*^lpr^ and *FcγRIIB^−/−^Yaa* (76, 77). While these data support a model in which TASL-dependent signaling in B cells represents a critical event in the initiation of autoreactive humoral responses, future studies are required to ascertain the relative contributions of the SLC15A4–TASL complex in distinct immune cell compartments. First, B cell specific deletion of complex components is needed to confirm and extend this hypothesis to other models (e.g., *Tlr7*^kik^), complementing our full-body knock-out investigation. Furthermore, deletion in pDCs, monocytes, and macrophages will reveal whether targeting the innate compartment would be sufficient to affect autoimmunity development. Last, inducible gene deletion after disease onset will further inform on the therapeutical potential of SLC15A4–TASL complex inhibition.

Across all models examined, SLC15A4–TASL complex deficiency resulted in a profound reduction of ABC populations. ABCs have emerged as a key pathogenic B cell subset in SLE through the production of inflammatory cytokines, enhanced antigen-presenting functions, and the differentiation into autoantibody secreting plasma cell and plasmablast (12, 44–46, 78, 79). In our study, disruption of the SLC15A4–TASL axis not only consistently prevented pathological ABC expansion across disease models but reduced ABC frequencies below those observed in WT controls. This effect extended to physiologically aged mice, revealing that TASL-dependent signaling is essential for both disease-associated and age-related ABC accumulation, in line with established role of TLR7 and IRF5 in promoting their differentiation (78, 80). Therefore, impairment of ABCs could represent a major mechanism underlying disease protection in TASL-deficient settings. The pronounced reduction of ABCs in aged mice raises the intriguing possibility that the SLC15A4–TASL axis may contribute to inflammaging, which should be addressed in future studies (81, 82). Of note, while this manuscript was in preparation, a study examining the impact of single TASL deficiency in the *Fas*^lpr^ (B6.MRL-Faslpr/J) model reported a similar requirement for TASL in ABC accumulation and disease progression (83).

Collectively, our findings demonstrating the central and nonredundant role of the SLC15A4–TASL complex across different models support its potential as a therapeutic target in SLE and related autoimmune conditions. Recent work by us and others demonstrated the druggability of this pathway, raising the possibility of selectively targeting IRF5 activation downstream of both TLR7 and TLR9 (30, 37). Such an approach may offer advantages over inhibition of TLR7/8 signaling or systemic blockade of type I interferons by affecting both TLR-mediated RNA and DNA responses or preserving IFN-mediated antiviral responses induced by other nucleic acid sensing pathways respectively. While future preclinical and clinical studies in SLE patients are required, the consistent protection observed across mechanistically distinct models suggests that targeting this pathway may be effective across the heterogeneous spectrum of human SLE, where diverse genetic and environmental triggers may often converge on endosomal nucleic acid–sensing pathways.

## Material and Methods

For detailed information, see *SI Appendix*, *Supporting Information Text*.

### Mice.

C57BL6/J strain (RRID:IMSR_JAX:000664) used as a WT control was originally obtained from Jackson Laboratory and bred and cohoused in the UNIL Epalinges animal facility. *Fas*^lpr^ mice (B6.MRL-Faslpr/J, RRID:IMSR_JAX:000482) were purchased from Charles River. *Tlr7*^kik^ mice (B6.Tlr7em2(Y264H)CgvAnu/Jcrick) were kindly provided by Carola G. Vinuesa from Francis Crick Institute, London, United Kingdom. All *Tlr7*^kik^ mice used in experiments were either hemizygote males (*Tlr7*^kik/Y^) or heterozygote females (*Tlr7*^kik/+^). *Dnase1l3*^KO^ mice were obtained from Taconic (TF2732) and backcrossed for more than 10 generations on C57BL/6 J. *Rag1*^KO^ mice on a C57BL/6J background were purchased from The Jackson laboratory (RRID:IMSR_JAX:034159). The *feeble* (*Slc15a4*^F/F^) mouse strain (C57BL/6J-Slc15a4m1Btlr/Mmjax, RRID:MMRRC_034296-JAX) was obtained from the Mutant Mouse Resource and Research Centre (MMRRC) at The Jackson Laboratory, an NIH-funded strain repository, and was donated to the MMRRC by Bruce Beutler, Ph.D., The Scripps Research Institute. Generation of *Tasl*^KO^ and *Tasl2*^KO^ mice by pronuclear injection of *Cas9* mRNA and respective gRNA was described previously (33). *Tasl*^DKO^ mice were obtained by crossing *Tasl*^KO^ and *Tasl2*^KO^. Experiments involving *Fas*^lpr^, *Tlr7*^kik^, and aged animals were performed under the guidelines of and with approval from the cantonal veterinary office of the canton of Vaud (Switzerland), license numbers VD3716x1, VD3779, and VD3779x1. Experiments involving *Dnase1l3*-*Rag1*^DKO^ mice were performed in accordance with the animal protocol approved by the local ethical committee and the French ministry of research (#11679).

### Flow Cytometry.

Single cell suspension splenocyte samples were blocked using anti-mouse CD16/32 antibody and stain with viability dye and antibodies detailed in *SI Appendix, Supporting Information Text*. Gating strategy for FC data analysis is provided in *SI Appendix*, Fig. S7.

### Autoantibodies ELISA.

ELISA experiments were carried out using mouse serum. Autoantibody detection was done using ELISA plates (ThermoFisher Scientific, 439454) coated with RNP/Sm antigen (2.5 μg/mL, Arotec diagnostics, ATR01), UltraPure™ Calf Thymus DNA (5 µg/mL, ThermoFisher Scientific, 15633019) or yeast RNA (2.5 µg/mL, ThermoFisher Scientific, AM7120G) overnight at 4 °C. Detailed protocol is described in *SI Appendix, Supporting Information Text*.

### Histology.

For histology imaging, longitudinally cut kidney, half a spleen and PBS perfused liver were harvested and fixed in 1% PFA at 4 °C for 1 h. Organs were then washed with PSB and incubated in 30% sucrose overnight at 4 °C, before embedding in Tissue-Tek OCT Compound (Sakura, 4583) and frozen. Details on staining protocol and antibodies used are described in *SI Appendix*, *Supporting Information Text*.

### ANA Staining.

Antinuclear antibodies assay was performed on mouse serum (dilution 1/300 for *Fas*^lpr^, 1/200 for *Tlr7*^kik^, 1/80 for *Dnase1l3*-*Rag1*^DKO^, 1/50 for 1 y aged mice) using Hep-2 ANA kit (NOVA Lite, 708100) and following the manufacturer’s instructions. The only changes were the replacement of goat anti-human IgG (in kit) with goat anti-mouse IgG AF488 (Jackson ImmunoResearch, 115-545-166), replacement of the mounting media provided in the kit with the Fluoroshield mounting medium (Sigma, F6182) and additional staining with DAPI (Invitrogen, R37606). Images were acquired using Zeiss Upright AxioVision.

### Adoptive Cell Transfer.

*Dnase1l3*-*Rag1*^DKO^ mice were obtained upon breeding and were then reconstituted with 10 × 10^6^ splenocytes from WT or *Tasl*^DKO^ mice, upon i.v injection into the retroorbital sinus.

### Statistics.

Data are presented as individual values, with mean ± SD where each data point represents a biological replicate (*n*). Statistical analyses were conducted using GraphPad Prism software (version 10.5.0). Statistical analysis was performed with one-way ANOVA using Dunnett’s or Tukey’s multiple comparisons test where appropriate for comparison of three and more groups. Two-way ANOVA with Šidák’s multiple comparisons test was used for specific pairwise comparison. The Mann–Whitney test was used for comparison of two groups. A *P* value of less than 0.05 was considered statistically significant.

## Supplementary Material

Appendix 01 (PDF)

## Data Availability

Study data are included in the article and/or *SI Appendix*.
