# Supplementary material for: The SLC15A4–TASL complex is essential for lupus development in mice
Source: Proc Natl Acad Sci U S A. 2026 Sep 1;123(36):e2618215123. doi: 10.1073/pnas.2618215123 (PMC13552680; doi:10.1073/pnas.2618215123)
Supplement: Supplementary file 1 [file pnas.2618215123.sapp.pdf]

PNAS

## Supporting Information for

# The SLC15A4–TASL complex is essential for lupus development in mice

Ales Drobek, Maeva Delacrétaz, Aliki Vasilakou, Marta Monguió-Tortajada, Léa Bernaleau, Maxime Dubois, Vanja Sisirak, and Manuele Rebsamen.

Manuele Rebsamen
Email: manuele.rebsamen@unil.ch

**This PDF file includes:**

Supporting Information text
Figures S1 to S7

## Supporting Information Text

### Detailed Materials and Methods

**Mice**. Mice were kept in individually ventilated cages in specific pathogen-free conditions with an irradiated standard rodent diet and water ad libitum in a facility with 12 h light/12 h dark cycle and stabile temperature 21 °C (± 1 °C) and 55 % (±10 %) humidity. Animals were euthanised using $CO_2$. This study examined male and female animals, and similar findings are reported for both sexes.

**Tissue and cell collection and purification**. Blood: Mouse blood was harvested upon submandibular puncture and collected in EDTA coated tube in order to obtain PBMC, and in a dry tube to obtain serum. At the terminal timepoint, blood was collected by cardiac puncture in freshly euthanized mice. Serum was isolated upon centrifugation at 2000g at 4°C or room temperature for 10min and frozen at -80 °C or -20 °C for further use. PBMC were isolated upon centrifugation at 300g at RT for 10min, followed by red blood cell lysis (1x ACK buffer: 150 mM $NH_4Cl$, 10 mM $KHCO_3$, 0.1 mM EDTA, pH 7.2) of the cell pellet for 5min at RT, and further stained for flow cytometry.
Spleen: Spleens were placed on PBS-humidified 70μm cell strainer, cut in two and one half mashed using a syringe plunger to obtain a single cell suspension. After 10min of centrifugation at 300g at RT, the cell pellet of splenocytes was incubated in presence of ACK buffer for 5min at RT and after washing step in PBS further used for flow cytometry.
For in vitro stimulation, splenic B cells were isolated using untouched magnetic separation (B cell isolation kit, Miltenyi, 130-090-862) on LS columns following manufacturer's protocol. Bone marrow derived macrophages (BMDM) were obtained by centrifugation of open bones (femurs and tibiae, 10.000 RPM for 1min) and, after red blood cell lysis in ACK buffer, cultivated in complete DMEM medium (10% FBS, 100 U/ml penicillin, 100 µg/mL streptomycin) supplemented with 10% M-CSF (L929 conditioned medium) for 7 days. Primary splenic B cells ($10x10^6$/mL in complete IMDM medium) or BMDM ($1x10^6$/mL) were plated and stimulated with 500μM guanosine (Sigma-Aldrich, G6264).

**Flow cytometry**. Single cell suspension splenocyte samples from *Fas*$^{lpr}$ and *Tlr7*$^{kik}$ experiments were blocked using TruStain FcX™ (anti-mouse CD16/32) antibody (93, Biolegend Cat # 101320), stained with LIVE/DEAD™ Fixable Near-IR kit (Invitrogen, Cat # L34975) and with the following anti-mouse antibodies from Biolegend: anti-CD3e AF488 (145-2C11, Cat # 100321), anti-CD4 BV421 (GK1.5, Cat # 100437), anti-CD8a BV510 (53-6.7, Cat # 100752), anti-CD44 BV605 (IM7, Cat # 103047), anti-CD62L AF700 (MEL-14, Cat # 104426), anti-CXCR5 PE (L138D7, Cat # 145503), anti-PD1 PE-Cy7 (29F.1A12, Cat # 135215), anti-B220 BV711 (RA3-6B2, Cat # 103255), anti-CD19 AF488 (6D5, Cat # 115521), anti-IgM BV421 (RMM-1, Cat # 406517), anti-IgD AF700 (11-26c.2a, Cat # 405725), anti-CD138 PE (281-2, Cat # 142503), anti-CD95 PE-Cy7 (SA367H8, Cat # 152617), anti-GL7 AF647 (GL7, Cat # 144606), anti-CD11c BV421 (N418, Cat # 117343), anti-CD23 PE/Dazzle 594 (B3B4, Cat # 101634), anti-CD21 AF647 (7E9, Cat # 123423), anti-I-A$^b$ PE-Cy7 (AF6-120.1, Cat # 116419), anti-CD86 AF647 (GL-1, Cat # 105019), anti-BST PE (927, Cat # 127010). Antibodies from Miltenyi: anti-Siglec-H PE and FITC (REA819, Cat # 130-112-296 and 130-112-142).
All blood PBMC were stained to verify *Dnase1l3-Rag1*$^{DKO}$ mice reconstitution upon splenocytes injection using the following anti-mouse antibodies from Biolegend: anti-CD4 FITC (RM4-4, Cat # 116004), anti-CD3 APC (17A2, Cat # 100236); anti-CD11b APC Cy7 (M1/70, Cat # A15390), anti-CD8 PECy7 (53-6.7, Cat # 100722), anti-B220 PE Dazzle (RA3-6B2, Cat # 103258) and anti-MHC II Pacific Blue (AF6-120.1, Cat # 116422). For the single cell suspension of the spleen, the Fc receptors were blocked using an anti CD16/CD32 antibody (93, Fisher Scientific Cat # 1963140) for 10min at 4°C and cells were then stained for viability using a Viadye (Cytek Biosciences). Then, cells were further stained using the following antibodies: anti-CD19 BUV737 (1D3, BD Cat # 612781), anti-CD62L BUV395 (MEL-14, BD Cat # 740218), anti-CD4 BUV494 (RM4-4, BD Cat # 741051), anti-CD11c BUV805 (GL1, BD Cat #746778), anti-CD44 BV510 (IM7, BD Cat #563114), anti-CXCR5 BV785 (L138D7, Biolegend Cat # 145523), anti Ly6C BV570 (HK1.4, Biolegend Cat # 128029), anti CD138 BV605 (281-2, Biolegend

Cat # 142531), anti-CD317 BV711 (927, BD Cat # 747604), anti-Ly6G Spark NIR 685 (1A8, Biologend Cat # 127665), anti-TCRb APC efluor 780 (H57-597, Fisher Scientific Cat # 47-5961-82), anti-CD23 AF700 (B3B4, Biolegend Cat # 101632), anti-MHC-II FITC (M5/114.15.2; Invitrogen Cat # 11-5321-82), anti-GL7 Pacific Blue (GL7, Biolegend Cat# 144614), anti-CD95 PE (SA367H8, Biolegend Cat # 152608), anti CD21/35 PE Cy7 (7 E9, Biolegend Cat # 123420), anti-B220 PE/Dazzle 594 (RA3-6B2, Biolegend Cat # 103258) and anti CD11b Red Fluor 710 (M1/70, Tonbo Cat # 35-0112-U500). Samples were acquired using Aurora (Cytek Technology), Symphony or LSRII (BD Biosciences) flow cytometers and analyzed using FlowJo software version 10 (Tree Star).

**Autoantibodies ELISA**. Coated plates with individual antigens were washed with PSB 1X, 0.01% Tween20 and blocked with PBS 1X, 2% BSA for 1 hour at RT. Blocking was follow by a washing step and samples were loaded in duplicates or triplicates (dilution 1/200 for *Fas*$^{lpr}$, 1/100 for *Tlr7*$^{kik}$ and *Dnase1l3-Rag1*$^{DKO}$) and incubated for 2 hours at RT. Plates were washed before adding secondary antibody (Goat anti-mouse IgG-HRP, Jackson Immunoresearch, 115-035-003), then incubated for 1 hour at RT. One last washing step was performed before TMB substrate (BD Biosciences, 555214) addition and stopping ($H_2SO_4$). Absorbance was measured at 450nm (subtracted by 570nm measurement) on a ThermoFisher Multiskan FC.
For longitudinal measurement of anti-dsDNA relative titers in sera: Nunc-Immuno maxisorp 96 well flat bottom plates were coated with 50 µL/well of 50 µg poly-L lysine (Sigma Aldrich) for 2h at RT, washed with PBS and coated with 50 µL/well of 10 µg/mL calf-thymus DNA (Calbiochem) in PBS, overnight at 4°C. Excess antigen was washed off using 2-3 washes with PBS-0,05% Tween. Antigen-coated plates were next blocked with 250 µL/well of PBS with 1% bovine serum, albumin (BSA) for 2h at RT. Blocked plates were washed once with PBS/0,05% Tween and coated with diluted serum (1/10 and 1/50) in PBS 1% BSA and incubated 2h at RT. Unbound serum antibodies were washed off with 3 washes of PBS-0,05% Tween. The bound IgG was detected using a 1/5000 dilution of goat anti-mouse IgG-AP-conjugate (Jackson Immunoresearch) diluted in PBS 1% BSA. Unbound secondary antibody was washed with PBS-0,05% Tween and developed using diethanolamine substrate buffer (ThermoFisher Scientific, 34064) and PNPP phosphatase substrate tablets (Sigma Aldrich, SIGMAS0942). For relative quantitation of antigen-specific IgG titers, serum from an anti-dsDNA positive animal was used as a standard using serial double dilutions. O.D. at the lowest serum dilution was arbitrarily assigned a value of 100 U/mL. Standard curve was plotted as O.D. at 405nm versus antigen concentration (U/mL). Relative IgG titers of samples were calculated using the straight-line equation and multiplied with the dilution factor.

**Histology**. After cutting, 5 µm sections were rehydrated in PBS for 3x 5 minutes, with a change of PBS between each time. Sections were blocked with assay mix (PBS 1X, 3% BSA, 0.1% Triton X-100 (Sigma, 93426)) for 2 hours at RT in a humid box, then stained overnight at 4°C with the following primary antibodies: Kidney: Rabbit anti-Podocin (Invitrogen, Cat # PA5-79757, 1/500); Spleen: Armenian Hamster anti-CD3ε (145-2C11, BioXCell, Cat # BE0001-1, 1/300), Rat anti-F4/80 (homemade supernatant, 1/4), Rat anti-CD169 (3D6.112, Biolegend, Cat # 142402, 1/500); Liver: Rat anti-CD45 (S18009D, Biolegend, Cat # 160302, 1/100). Slides were washed 3 times with PBS, then incubated 45 minutes at RT in a humid box with the following secondary antibodies diluted in assay mix: Kidney: Goat anti-mouse IgG AF488 (Jackson ImmunoResearch, Cat # 115-545-166, 1/750), Goat anti-mouse IgM AF488 (Jackson ImmunoResearch, Cat # 115-545-075, 1/750), Goat anti-Rabbit AF568 (Invitrogen, Cat # A-11011, 1/750); Spleen: Goat anti-Armenian Hamster IgG AF488 (Jackson ImmunoResearch, Cat # 127-545-160, 1/500), Donkey anti-Rat IgG AF647 PLUS (Invitrogen, Cat # A48272, 1/500); Liver: Goat anti-Rat IgG AF555 (Invitrogen, Cat # A48263, 1/1000). Slides were washed 3 times in PBS and spleen sections only were blocked with 4% normal rat serum (Sigma, R9759) before staining with the third antibody: Spleen: Rat anti-B220 BV421 (RA-3-6B2, Biolegend, Cat # 103251, 1/500). Slides were again washed and stained either with DAPI (Invitrogen, R37606, 1/5) or with DRAQ5 (BioStatus, DR50200, 1/2000) for 5 minutes at RT before three final washes with PBS 1X. Slides were then mounted with coverslips using Fluoroshield mounting medium (Sigma, F6182).

Images were acquired using Hamamatsu Nanozoomer. IgG or IgM deposition in glomeruli was quantified by mean fluorescence intensity (MFI) using FIJI software (version 2.16.0) by selecting identical area sizes over 10 glomeruli per kidney section. Data are displayed as mean of MFI ratio (IgG/Podocin or IgM/Podocin) per mouse. Liver images were analysed by QuPath (version 0.6.0).

**Immunoblotting**. Cells were lysed in RIPA lysis buffer (25 mM Tris, 150 mM NaCl, 0.5% NP-40, 0.5% deoxycholate (w/v) and 0.1% SDS (w/v), pH 7.4) supplemented with Roche EDTA-free protease inhibitor cocktail (one tablet for 50 mL), Halt phosphatase inhibitor cocktail (Thermo Fisher Scientific,78420) and Benzonase (Merck, 71205), 10 min on ice. Protein concentration was quantified with BCA (ThermoFisher Scientific) using BSA as a standard. Cell lysates were resolved by regular or Phos-tag-containing (20 µM, WAKO Chemicals, WA3 304-93521) SDS–PAGE. After electrophoresis, Phos-tag-containing SDS–PAGE were soaked in transfer buffer with 10 mM EDTA for 3x 10 min, rinsed 10 min in transfer buffer without EDTA and blotted to nitrocellulose membranes (Amersham, Glattbrugg, Switzerland). Membranes were blocked with 5% non-fat dry milk in TBST and probed with the indicated antibodies. Following primary antibodies were used: SAPK/JNK (9252), phospho-SAPK/JNK Thr183/Tyr185 (4668), NF-κB p65 (8242), phospho-NF-κB p65 Ser536 (3033), IκBα (4814), phospho-IκBα Ser32 (2859), all from Cell Signalling and used at 1/1000 dilution. IRF5 (ab181553, 1/1000) was from Abcam and GAPDH (sc-365062, 1/1000) was from Santa Cruz. Primary antibody binding was detected with anti-mouse-HRP secondary antibodies (115-035-003) or anti-rabbit-HRP secondary antibodies (111-035-003), from Jackson ImmunoReasearch, using the WesternBright ECL HPR substrate (Advansta, K-12045-D50). When multiple antibodies were used, equal amounts of samples were loaded on multiple SDS–PAGE gels and western blots were sequentially probed with a maximum of two antibodies.

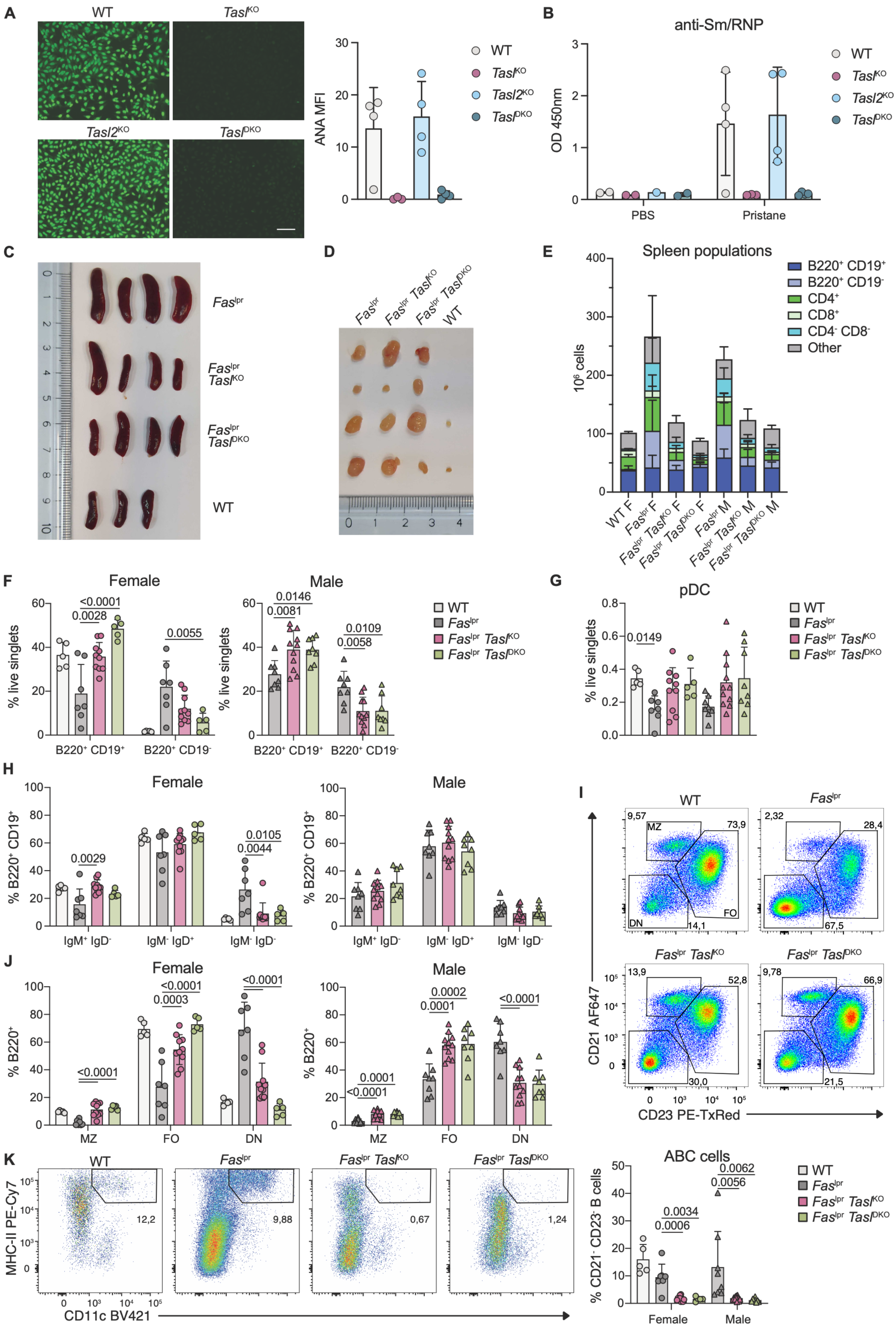


**Fig. S1. Amelioration of disease in *Fas*lpr mice by TASL deletion.**

(***A***) Serum anti-nuclear antibodies (ANA, 1/300) assessed by Hep-2 immunofluorescence. Representative images and MFI quantification are shown. Scale = 100 $\mu$m**.** (***B***) Autoantibody levels against Sm/RNP self-antigen in sera 4 mo after pristane i.p. injection measured by ELISA. (***C-D***) Spleen (*C*) and cervical lymph node (*D*) size comparison between genotypes in 8 mo old mice. (***E***) Mean proportion of main immune cell types in spleen. (***F***) Spleen analysis of double ($B220^+$ $CD19^+$) and single ($B220^+$ $CD19^-$) positive B cells. (***G***) Frequency of splenic pDCs ($CD11c^+$ $B220^+$ Siglec-$H^+$). (***H***) Expression of IgD vs IgM in splenic B cells ($B220^+$ $CD19^+$). (***I-J***) Representative dot-plot showing gating for marginal zone (MZ, $CD21^+$ $CD23^{low}$), follicular (FO, $CD21^{int}$ $CD23^+$) and double negative ($CD21^-$ $CD23^-$) splenic B cells ($B220^+$) (*I*) with quantification (*J*). (***K***) Representative dot-plot showing gating for ABC cells ($B220^+$ $CD21^-$ $CD23^-$ MHC-$II^{high}$ $CD11c^+$) in spleen with quantification. Data in all graphs are shown as means ± SD. Statistical analysis was performed with one-way ANOVA using Dunnet's multiple comparisons test to *Fas*$^{lpr}$ mice. Circle datapoints represent females and triangles males. (*A-B*) *n*=3-4, (*E-H, J-K*) *n*=5-11 per genotype.

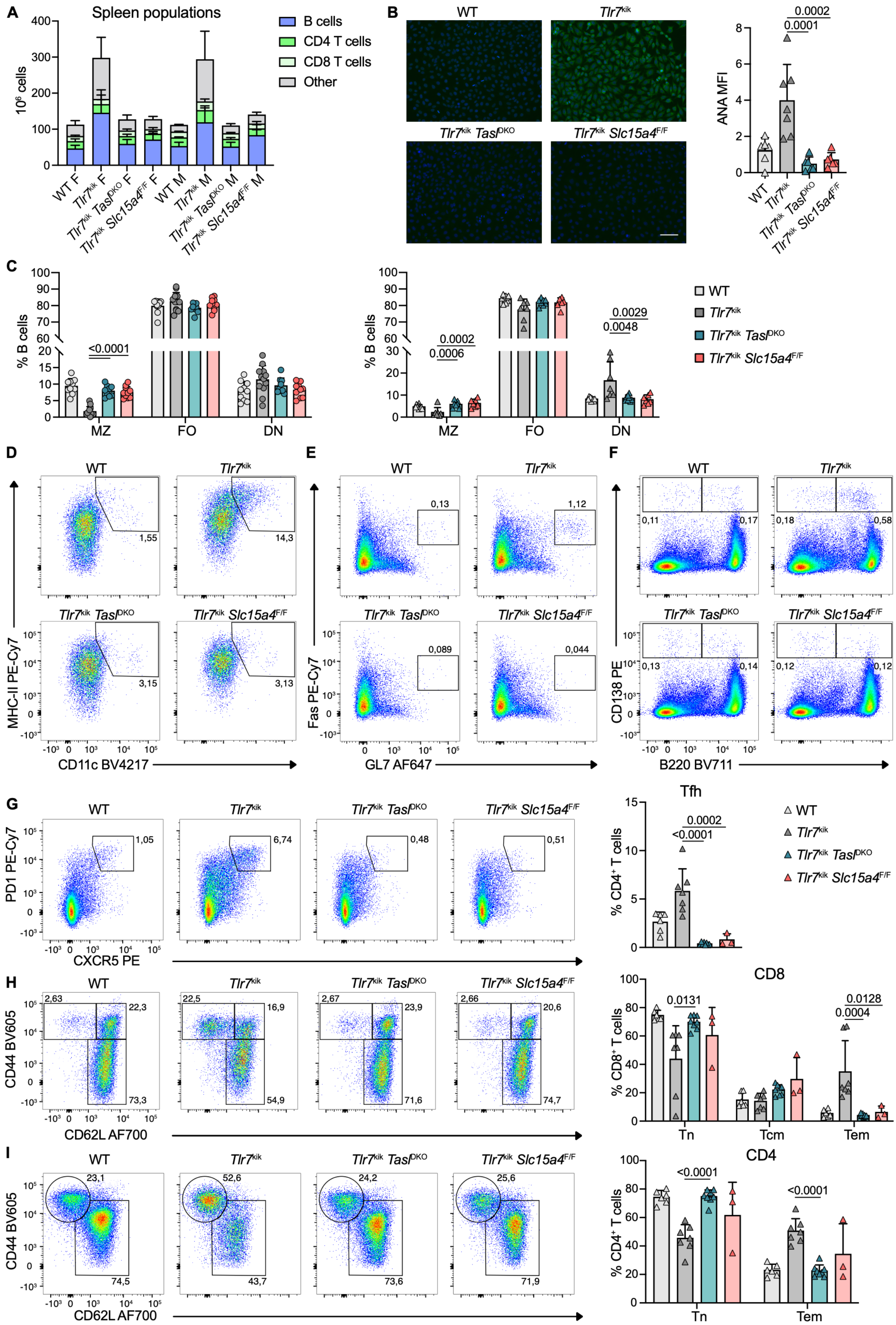


**Fig. S2. Effect of SLC15A4-TASL complex deficiency on SLE phenotype development in *Tlr7*kik mice.**

(***A***) Mean proportion of main immune cell types in spleen of 12 wk old mice. (***B***) Serum anti-nuclear antibodies (ANA, 1/200) assessed by Hep-2 immunofluorescence. Representative images and MFI quantification are shown. (***C***) Percentage of marginal zone (MZ, $CD21^{+}$ $CD23^{low}$), follicular (FO, $CD21^{int}$ $CD23^{+}$) and double negative ($CD21^{-}CD23^{-}$) splenic B cells ($B220^{+}$ $CD19^{+}$). (***D***) Representative dot-plot showing gating for ABC cells ($CD19^{+}$ $B220^{+}$ $CD21^{-}$ $CD23^{-}$ MHC-$II^{high}$ $CD11c^{+}$) in spleen. (***E-F***) Representative dot-plot showing gating for germinal center ($Fas^{+}$ $GL7^{+}$) (*E*) and long-lived plasma cells ($CD138^{+}$ $B220^{-}$) and short-lived plasmablasts ($CD138^{+}$ $B220^{+}$) (*F*) in splenic B cells ($B220^{+}$ $CD19^{+}$). (***G-I***) Dot-plot of splenic T cells ($CD3\varepsilon^{+}$) showing gating of $CD4^{+}$ T follicular helper (Tfh, $CXCR5^{+}$ $PD1^{+}$) cells (*G*), $CD8^{+}$ naïve (Tn, $CD62L^{+}$ $CD44^{-/low}$), central memory (Tcm, $CD62L^{+}$ $CD44^{+}$) and effector memory (Tem, $CD62L^{-}$ $CD44^{+}$) T cells (*H*) and $CD4^{+}$ naïve (Tn) and effector memory (Tem) T cells (*I*). Statistical analysis was performed with one-way ANOVA using Tukey's multiple comparisons test, means ± SD. Circle datapoints represent females and triangles males. (*A*, *C*) *n*=8-12, (*B*) *n*=6-7, (*G-I*) *n*=3-8 per genotype.

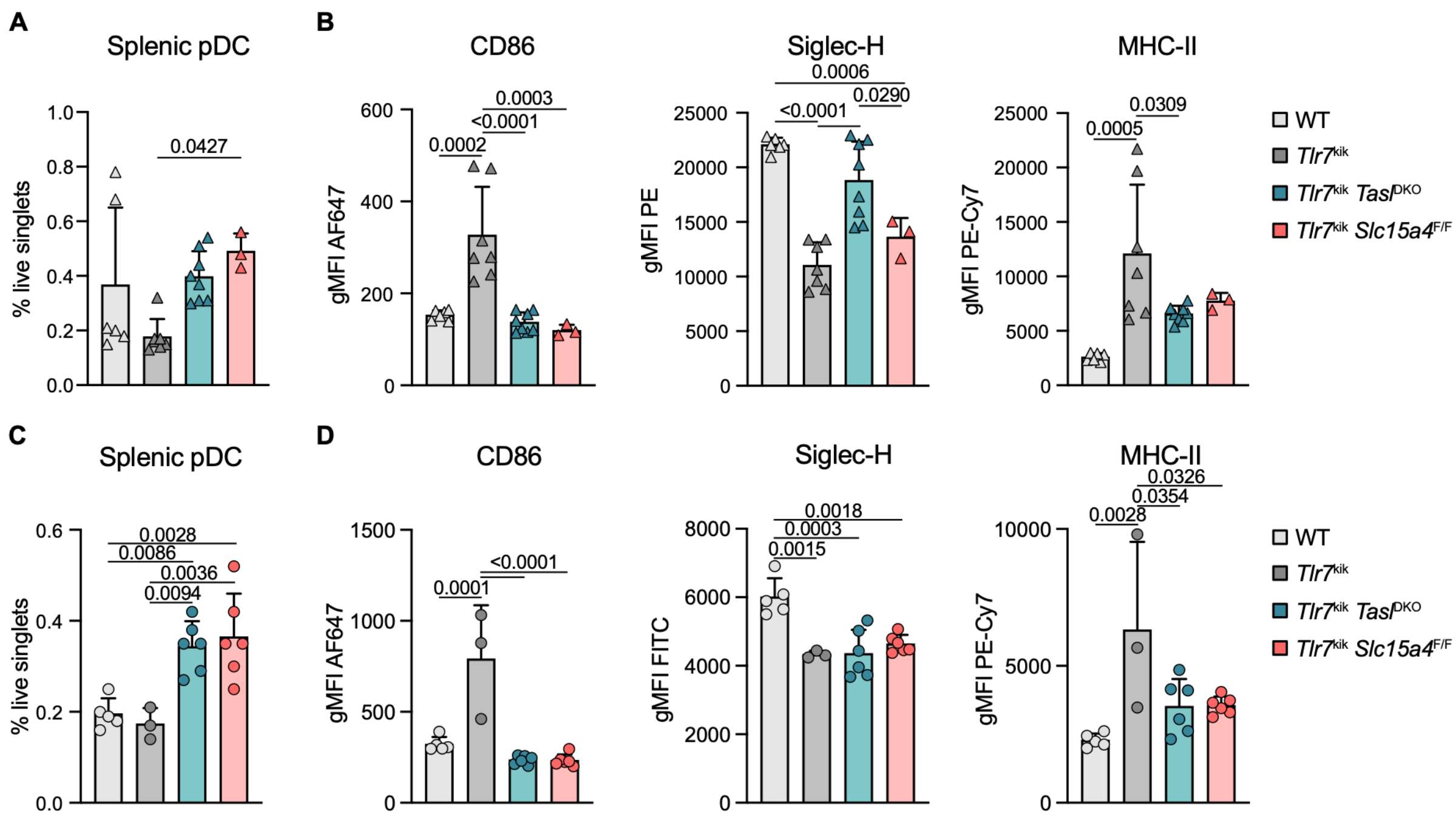


**Fig. S3. Splenic pDCs have activated phenotype in *Tlr7*$^{kik}$ mice.**
(***A, C***) Frequency of pDCs (CD11c$^{+}$ Siglec-H$^{+}$) among live singlet cells. (***B, D***) Expression level of CD86, Siglec-H and MHC-II on pDCs measured as gMFI. Statistical analysis was performed with one-way ANOVA using Tukey's multiple comparisons test, means ± SD. Circle datapoints represent females and triangles males. (*A-B*) *n*=3-8, (*C-D*) *n*=3-6 per genotype.

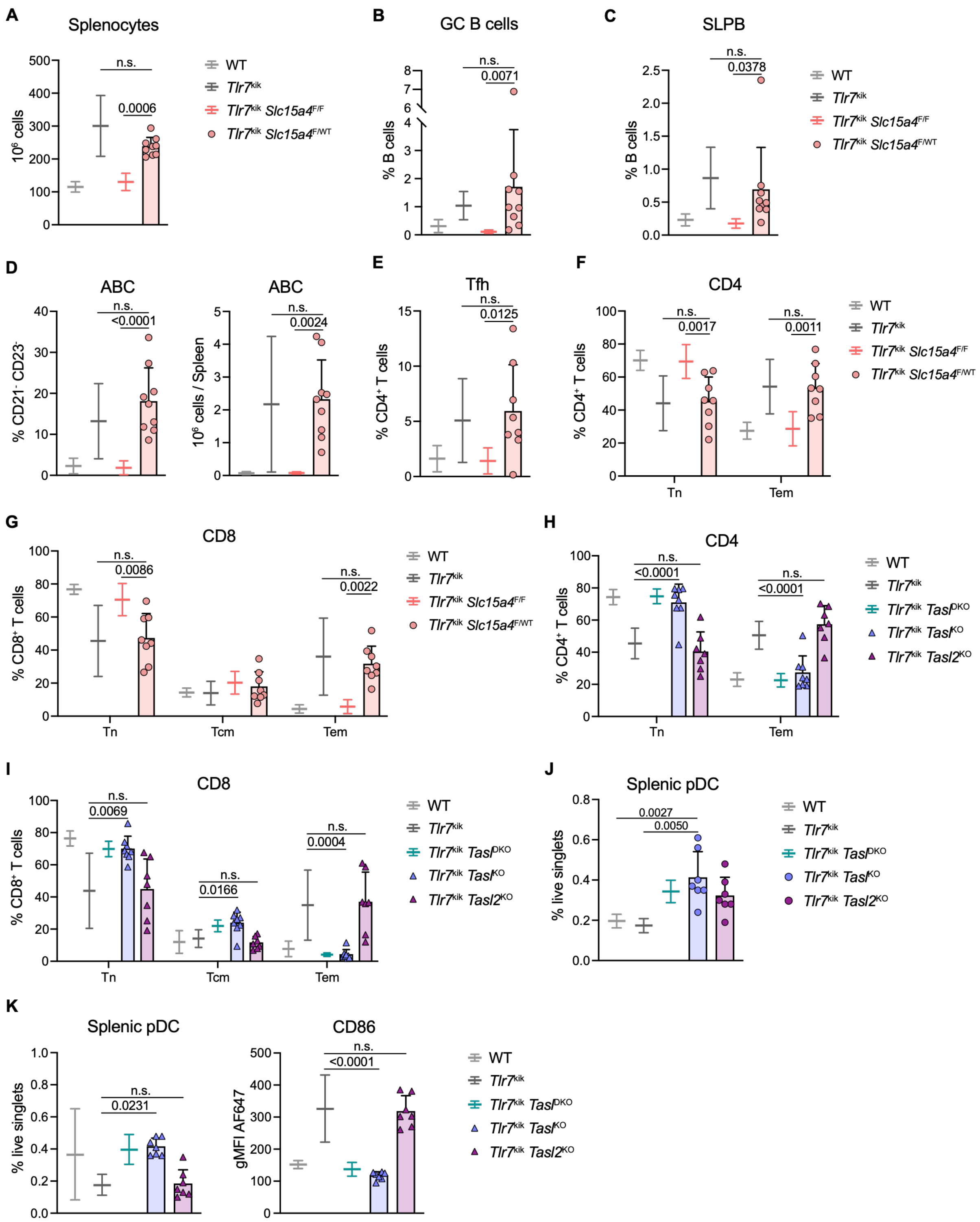


**Fig. S4. *Slc15a4*$^{F/WT}$ is insufficient to mediate protection from *Tlr7*$^{kik}$ gain of function.**
(***A***) Spleen cellularity at 12 wk of age. (***B-D***) Flow cytometry analysis of splenic B cells (CD19$^+$ B220$^+$) showing percentage of germinal center (Fas$^+$ GL7$^+$) (*B*) and plasmablasts (B220$^+$ CD138$^+$) (*C*) and percentage of ABC cells (MHC-II$^{high}$ CD11c$^+$) in DN B cells (CD21$^-$ CD23$^-$) and total number per spleen (*D*). (***E-I***) Flow cytometry analysis of splenic T cells (CD3ε$^+$) showing percentage of CD4$^+$ follicular helper (Tfh, CXCR5$^+$ PD1$^+$) T cells (*E*), percentage of CD8$^+$ naïve (Tn, CD62L$^+$ CD44$^{-/low}$), central memory (Tcm, CD62L$^+$ CD44$^+$) and effector memory (Tem, CD62L$^-$ CD44$^+$) T cells (*G*, *I*) and percentage of CD4$^+$ naïve (Tn) and effector memory (Tem) T cells (*F*, *H*). (***J-K***) Frequency of splenic pDCs (CD11$^+$ Siglec-H$^+$) and their CD86 expression shown as gMFI. In panels *A-K*, data on WT, *Tlr7*$^{kik}$, *Tlr7*$^{kik}$ *Slc15a4*$^{F/F}$ and *Tlr7*$^{kik}$ *Tasl*$^{DKO}$ mice from Fig. 2 and Fig. S3 are shown as lines representing mean

with ± SD to facilitate comparison with *Tlr7*$^{kik}$ *Tasl*$^{KO}$, *Tlr7*$^{kik}$ *Tasl2*$^{KO}$ and *Tlr7*$^{kik}$ *Slc15a4*$^{F/WT}$ mice. Statistical analysis was performed with one-way ANOVA using Tukey's multiple comparisons test, means ± SD. Circle datapoints represent females and triangles males. (*A*-*G*) *n*=8-9, (*H*-*I*) *n*=7-8, (*J*-*K*) *n*=7 per genotype.

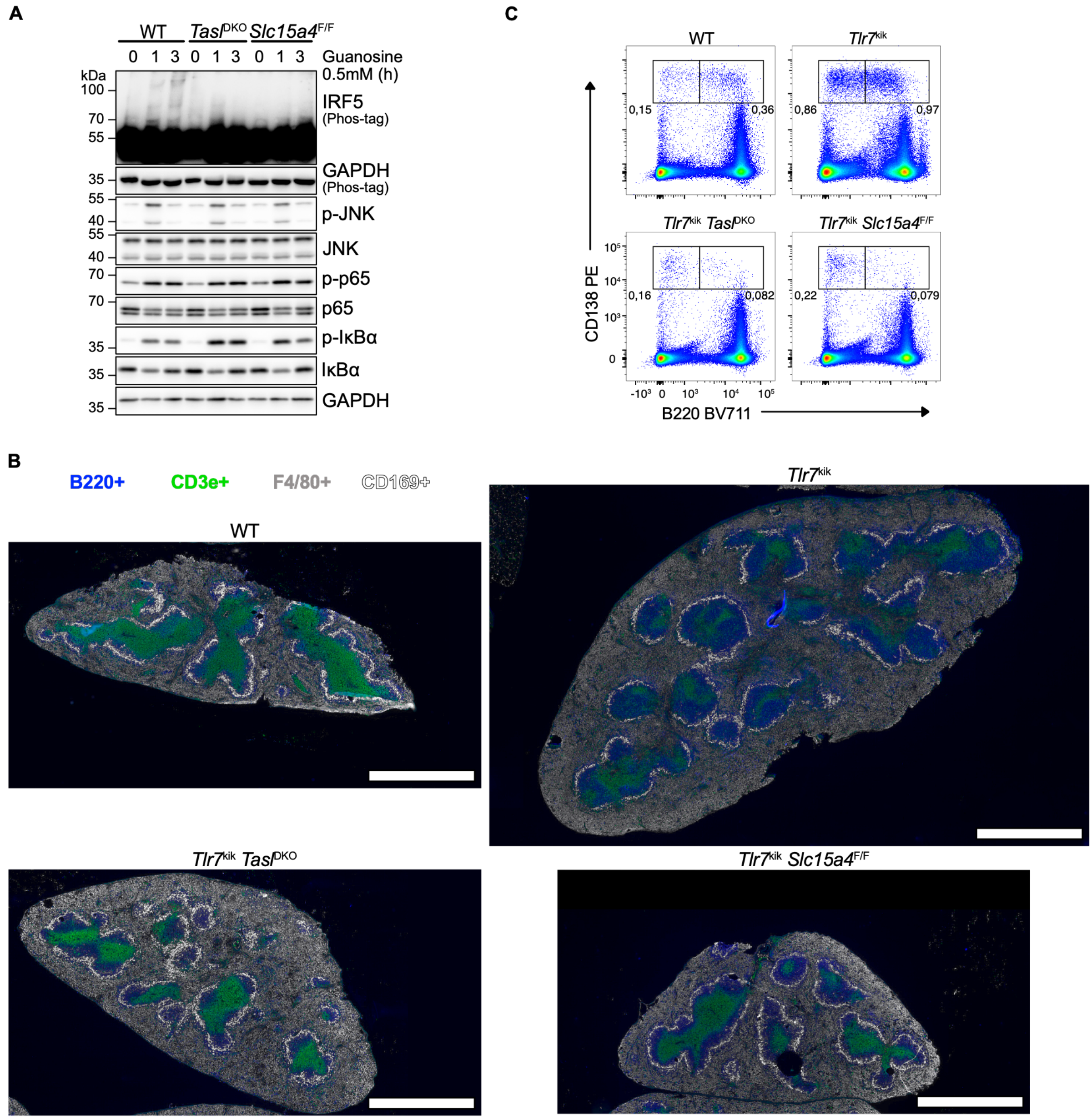


**Fig. S5. Loss of the SLC15A4-TASL complex prevents splenomegaly in *Tlr7*$^{kik}$ aged mice.**
**(*A*)** WT, *Tasl*$^{DKO}$ and *Slc15a4*$^{F/F}$ bone marrow-derived macrophages were stimulated with guanosine (500μM) and analysed by immunoblotting. Data representative of two independent experiments. **(*B*)** Representative microscopy images of spleen sections for each genotype stained with anti-B220 (B cells), anti-CD3ε (T cells), anti-F4/80 (red pulp macrophages) and anti-CD169 (marginal zone macrophages). Scale 1 mm. **(*C*)** Representative dot-plot gating of long-lived plasma cells (LLPC, CD138$^{+}$ B220$^{-}$) and short-lived plasmablasts (SLPB, CD138$^{+}$ B220$^{+}$).

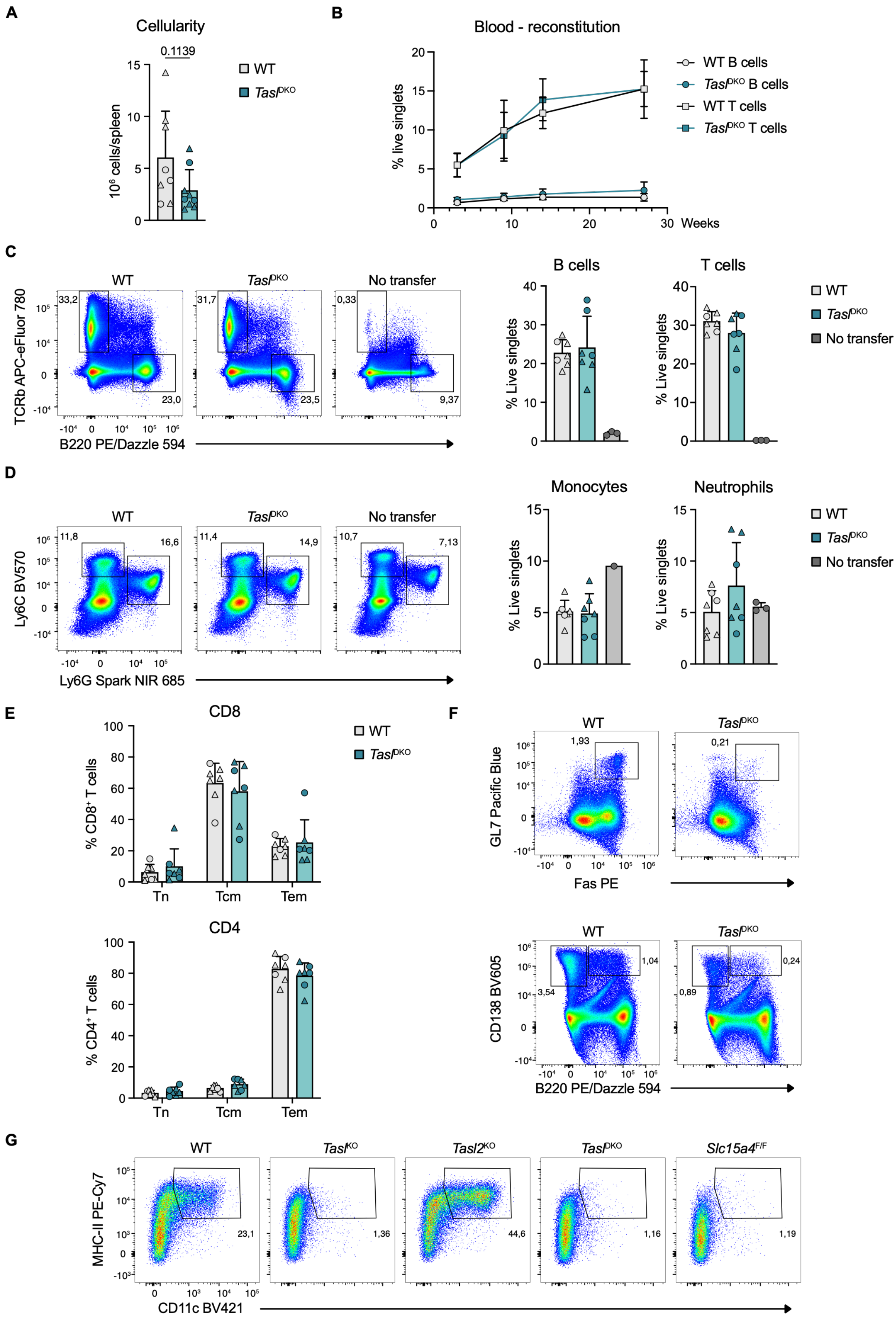


**Fig. S6. WT and *Tasl*$^{DKO}$ lymphocyte reconstitution in lymphopenic *Dnase1l3* deficient mice.**

(***A***) Spleen cellularity in *Dnase1l3-Rag1*$^{DKO}$ mice reconstituted with WT or *Tasl*$^{DKO}$ splenocytes analysed 7 mo after adoptive transfer. (***B***) Longitudinal analysis of B ($CD19^+$ $B220^+$) and T ($CD3\varepsilon^+$ $CD19^-$) cell reconstitution in blood of *Dnase1l3-Rag1*$^{DKO}$ mice. (***C***) Reconstitution analysis of B ($CD19^+$ $B220^+$) and T ($TCR\beta^+$ $B220^-$) cells in spleen of *Dnase1l3-Rag1*$^{DKO}$ mice. Representative dot plots with quantification are shown. (***D***) Monocytes ($Ly6C^+$) and granulocytes ($Ly6G^+$) percentage in spleen ($TCR\beta^-$ $B220^-$ cells) with and without reconstitution. Representative dot plots with quantification are shown. (***E***) $CD8^+$ and $CD4^+$ T cell phenotype 7 months after adoptive transfer of splenocytes. Naïve (Tn, $CD62L^+$ $CD44^{-/low}$), central memory (Tcm, $CD62L^+$ $CD44^+$) and effector memory (Tem, $CD62L^-$ $CD44^+$) T cells. (***F***) Representative dot plots with gating of GC B cells ($Fas^+$ $GL7^+$ $CD19^+$ $B220^+$) and plasma cells (PC, $CD138^+$ $B220^-$) or plasmablasts (PB, $CD138^+$ $B220^+$) in live singlets. (***G***) Representative dot plots with gating of splenic ABC B cells ($CD19^+$ $B220^+$ $CD21^-$ $CD23^-$ MHC-$II^{high}$ $CD11c^+$) in 1-y-old female mice. Circle datapoints represent female and triangles male *Dnase1l3-Rag1*$^{DKO}$ recipient mice (*A*, *C-E*). Statistical analysis was performed with Mann-Whitney test (*A*, *C-E*), means ± SD. (*A-B*) n=8-9, (*C-E*) n=7 per genotype.

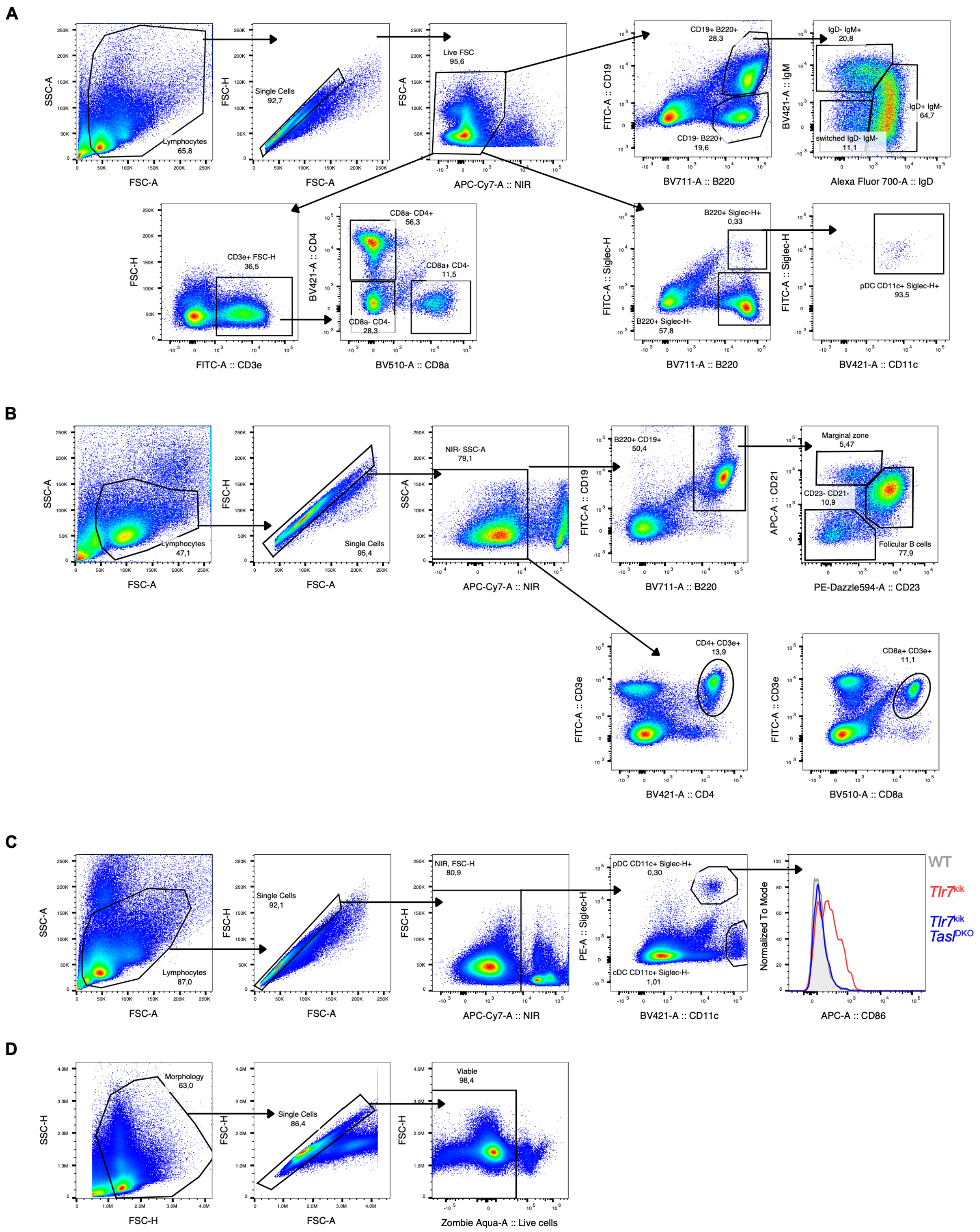


**Fig. S7. Gating strategy for flow cytometry data.**

(***A***) Gating in *Fas*[lpr] model analysis. Related to data presented in Fig. 1 and S1 (***B-C***) Gating in *Tlr7*[kik] model analysis. Related to data presented in Fig. 2-4 and S2-5. (***D***) Gating in *Dnase1l3* model analysis. Related to data presented in Fig. 5 and S6.
